# The Interplay Between Immunological Status and Gut Microbial Dysbiosis in the Development of the Symptoms of Irritable Bowel Syndrome: A Systematic Review with Meta-Analysis

**DOI:** 10.1007/s10620-025-09360-w

**Published:** 2025-09-03

**Authors:** Tasnia Ahmed, Daniel A. Lemberg, Andrew S. Day, Steven T. Leach

**Affiliations:** 1 School of Clinical Medicine, Faculty of Medicine and Health, UNSW Sydney, Kensington Campus, Sydney, NSW 2052 Australia; 2 Department of Gastroenterology, Sydney Children’s Hospital, Randwick, NSW 2031 Australia; 3 Cure Kids Chair of Paediatric Research, Department of Paediatrics, University of Otago, Christchurch, New Zealand; 4 School of Clinical Medicine, Faculty of Medicine and Health, UNSW Sydney, Kensington Campus, Sydney, NSW 2052 Australia

**Keywords:** Inflammation, Inflammatory marker, Irritable bowel syndrome, Gut microbiota

## Abstract

**Purpose:**

Irritable bowel syndrome (IBS) is a chronic disorder of gut–brain interaction (DGBI) characterized by recurrent abdominal pain and altered bowel habits. Treatment typically focuses on symptom management without addressing underlying causes. This systematic review and meta-analysis aimed to explore the association between inflammatory markers and gut microbiome changes in individuals with IBS.

**Methods:**

A systematic search of PubMed, Scopus, EMBASE, and CINAHL databases was conducted in June 2024, identifying 41 studies that compared inflammatory markers and gut microbial composition in patients with IBS versus healthy controls. Meta-analysis was performed using a random-effects model, reporting standard mean differences (SMD) for inflammatory markers and mean differences (MD) for microbiome data, with 95% confidence intervals (CI).

**Results:**

The results showed significantly elevated pro-inflammatory markers in individuals with IBS, including Interleukin (IL)-6 (SMD =  − 2.64), Tumor Necrosis Factor (TNF)-α (SMD =  − 1.97), platelet-to-lymphocyte ratio (PLR, SMD =  − 0.98), and fecal calprotectin (FC, SMD =  − 0.42), while IL-10 was lower (SMD = 2.00). Microbiome analysis revealed increased Bacteroidetes (MD =  − 15.92) and decreased Firmicutes (MD = 16.85) in people with IBS.

**Conclusion:**

These findings suggest that inflammation and gut microbial imbalance contribute to IBS, warranting further studies on their interplay and impact on the typical symptoms seen in IBS.

**Supplementary Information:**

The online version contains supplementary material available at 10.1007/s10620-025-09360-w.

## Introduction

Irritable bowel syndrome (IBS) is a disorder of gut–brain interaction (DGBI) that is generally associated with abdominal pain followed by a change in stool frequency and form, with unknown etiology [1–3]. Management of IBS does not rely on understanding the underlying pathophysiology; instead, it is focused on limiting symptoms [1, 4]. Mucosal inflammation and immune activation, alteration in the gut microbiome, changes in intestinal permeability, dysregulation of the hypothalamic–pituitary–adrenal (HPA) axis, changes in bile acid metabolism, food sensitivity, carbohydrate malabsorption, and infection have all been proposed as putative causes of symptoms exhibited by people with IBS [1, 5–11].

As the immune response is proposed to contribute to the symptoms of IBS, inflammatory markers have also been proposed as biomarkers for IBS diagnosis. [2, 3, 7, 8, 10–38]. Inflammatory cells, including mast cells and T-lymphocytes in the colon, were found to be increased in people with IBS [29, 39]. Intestinal mast cells are located near mucosal innervation [13]. Mast cell degranulation releases molecules that can excite nearby nerves, causing visceral hypersensitivity and disrupted gut–motor function [40, 41]. This provides a plausible etiology for symptoms, including abdominal pain, diarrhea, and constipation [13, 40–43]. However, there are further studies that conversely report decreased mast cells and T cells in IBS [14].

In addition to intestinal inflammation, an imbalance in the gut microbiota has been implicated in the development of IBS [25, 44]. Gut infection and bacterial overgrowth, which can alter the gut microbiota, are thought to be potential predisposing factors [25, 45] and have been associated with the severity of IBS symptoms [46]. Fecal bacterial communities derived from individuals with IBS, when introduced into mice models, can introduce dysfunctionality, including visceral hypersensitivity, gut epithelial hyperpermeability, and intestinal dysmotility [23]. There have also been reports of high *Firmicutes/Bacteroidetes* ratios in some individuals with IBS [2, 47, 48], as well as increased *Bacteroides* and decreased *Methanobacteriales, Prevotella, Bifidobacterium*, and *Faecalibacterium prausnitzii* [34, 45, 46]. Some studies report the association of IBS symptoms with microbial richness, the presence of methanogens, and the increase in Clostridiales or *Prevotella* species [27, 46].

Gut microbiota can induce immune activation via lipopolysaccharide (LPS) or short-chain fatty acids derived from bacteria [49–52]. In addition, gut bacteria can modify bile acids; such modifications are also linked with IBS symptoms [27, 53]. Furthermore, microbial changes in composition often enhance the bile acid modifications by affecting bile acid metabolism, increasing bile secretion [53, 54]. The gut microbial community is also not limited to bacteria and consists of a smaller number of other microbes, including fungi. Fungi can also induce host immune dysfunction, as the administration of antifungal drugs has been reported to induce recovery from visceral hypersensitivity in rats [55].

While there is some evidence linking immune activation with gut microbial imbalances in IBS, there is no previous systematic assessment of these two factors to explore how immune dysregulation may influence gut microbiota composition and contribute to symptoms in IBS. This manuscript fills that gap by providing the first systematic review and meta-analysis that evaluates immune and microbial factors independently, while also reviewing existing studies on how immune dysregulation affects gut microbiome changes, offering new insights into their complex interactions in IBS pathophysiology. This systematic review aimed to establish evidence of inflammatory and immune marker activation, intestinal microbiota dysbiosis, and associations with immune activation and microbiota dysbiosis in IBS.

## Methods

### Search Strategy

This systematic review was registered at the initial stage on PROSPERO, an international prospective register of systematic reviews (Registration ID CRD42024577724; 18 August 2024). The systematic review was planned and conducted by following the Preferred Reporting Items for Systematic Reviews and Meta-Analyses (PRISMA) Guideline [56].

A literature search was performed using PubMed, EMBASE Excerpta Medica Database, Scopus, and CINAHL (Cumulative Index to Nursing and Allied Health Literature) databases. The search terms for PubMed were ‘irritable bowel syndrome,’ ‘inflammation,’ ‘children’ connected by AND, and ‘inflammatory marker in irritable bowel syndrome’ as a title search, and combining all the search results. The search included ‘gut microbiome or gut bacteria or intestinal bacteria AND inflammation AND irritable bowel syndrome’ to find papers related to intestinal bacteria linked with low-grade intestinal inflammation among IBS and healthy controls. Search terms for Scopus included ‘inflammatory AND markers AND in AND irritable AND bowel AND syndrome.’ Filters were used to limit search results to medicine, articles, published, English, all open access, and seven keywords (irritable colon, irritable bowel syndrome, biomarkers, interleukin 6, interleukin 8, interleukin 10, and fecal calprotectin). In the same database, searches were conducted for *(TITLE-ABS-KEY (irritable AND bowel AND syndrome OR ibs)) AND (TITLE-ABS-KEY (gut AND inflammation)) AND (TITLE-ABS-KEY (gut AND microbiota)). The EMBASE database included the search terms: ‘Inflammatory marker$.mp.’ and ‘irritable bowel syndrome.mp.’ and combined both terms. The search term for bacteria linked with low-grade inflammation in IBS included ‘gut bacteria and irritable bowel syndrome$.mp.’ The fourth and final database was CINAHL with search terms ‘Inflammatory markers or inflammation AND Irritable bowel syndrome or IBS’ (filters were applied for: academic journal, language-English), and ‘irritable bowel syndrome or IBS’ AND ‘gut microbiome or gut microbiota or gut bacteria’ AND ‘inflammation.’ During the search, no limit was placed on the year of publication. Some studies were included by citation search from the included articles while reading for data collection. The literature search was conducted between June 2024 and July 2024.

### Inclusion–Exclusion Criteria

Inclusion criteria were original research articles, any age, any gender, including healthy controls, and comparing inflammatory markers and gut bacteria between IBS [and/or IBS subgroups: IBS-D (diarrhea predominant), IBS-C (constipation predominant), IBS-M (mixed), IBS-A (IBS alternating bowel habits), IBS-U (unsubtyped/unspecified) PIIBS (post-infectious IBS), NPIIBS (non-post-infectious IBS)] and controls. Studies that included review articles, conference abstracts, animal models, no healthy controls, focusing on other gastrointestinal diseases and comparing them with controls, treatments, full article not accessible, languages other than English, wrong study design (the studies which were designed based on completely different aim which does not fit with the objective of this review), and wrong data type/measure (studies with data types or measurements that were incompatible with those of the included studies) were excluded. All articles with similar measurement types of the data were included in this review; wrong patient population (e.g., when only post-infectious IBS was considered), and wrong indication (where other specific indication of IBS was studied instead of inflammatory markers or gut microbiome) not related to low-grade inflammation (articles which did not aim to study the inflammatory conditions of IBS) were excluded from the current study.

### Study Selection

The search reference list was imported into Covidence (an online tool) for screening (https://www.covidence.org) [57]. After the automatic removal of duplicates, 1680 articles remained, with 6 added from the citation search. Two independent reviewers screened the reference list by title, abstract, and keyword for matches to the inclusion/exclusion criteria.

### Data Collection

Data were extracted from applicable studies into an Excel sheet (Microsoft, version 2408, Build 17,928.20080). Extracted data included author, year of publication, country of origin, study duration, age mean, number of participants, male-to-female ratio, detection markers, sample type, the result for IBS and control, and *p* value for IBS vs. control. Data on markers directly or indirectly linked to subclinical inflammation in IBS, compared to healthy controls, were also entered into the Excel spreadsheet. Some studies reported age as a range or median; in this instance, mean age was calculated using the Meta-analysis Accelerator online tool (https://meta-converter.com) [58]. Additional data reporting values as median and range were also converted to mean and standard deviation to get a consistent value for meta-analysis using the Meta-analysis Accelerator online tool [58]. This is an automated data conversion tool used for meta-analysis to incorporate similar data types.

### Statistical Analysis

Meta-analysis was conducted when two or more studies reported compatible metrics. For meta-analysis, RevMan (Review Manager) Web, powered by Cochrane, was used (https://revman.cochrane.org/info). Values were reported as mean difference (for bacterial analysis) and standard mean differences (for inflammatory markers) with a 95% confidence interval (CI) calculated using DerSimonian and Laird’s random-effects method. For bacterial analysis, the mean difference showed no heterogeneity, whereas the standard mean difference showed high heterogeneity; therefore, mean differences for bacterial analysis were utilized. A statistically significant *p* value was set as less than 0.05. Statistical heterogeneity was calculated as *I*^2^, where < 40% is not important, 30–60% indicates moderate heterogeneity, 50–90% represents substantial heterogeneity, and 75–100% indicates considerable heterogeneity. Subgroup analysis by age, study type, and IBS type was performed for different inflammatory markers to explore possible causes of heterogeneity. A sensitivity test was performed for high heterogeneity to examine the influence of excluding or including studies within subgroups to evaluate the reliability of subgroup findings.

### Risk of Bias Assessment

Quality assessment of all the included studies was performed using the Cochrane risks of bias tool ROBINS-I (https://methods.cochrane.org/robins-i). Seven domains of questions were answered to determine the overall risk of bias as low, moderate, serious, or critical. The seven domains of the tool are as follows—(1) bias due to confounding, (2) bias due to selection of participants, (3) bias in classification of interventions, (4) bias due to deviations from intended interventions, (5) bias due to missing data, (6) bias in measurement of outcomes, and (7) bias in selection of the reported result. The online ‘robvis’ tool (https://www.riskofbias.info/welcome/robvis-visualization-tool) was used to visualize the risk-of-bias results of the articles included in the systematic review [59].

## Results

### Search Results and Study Selection

**Fig. 1:**
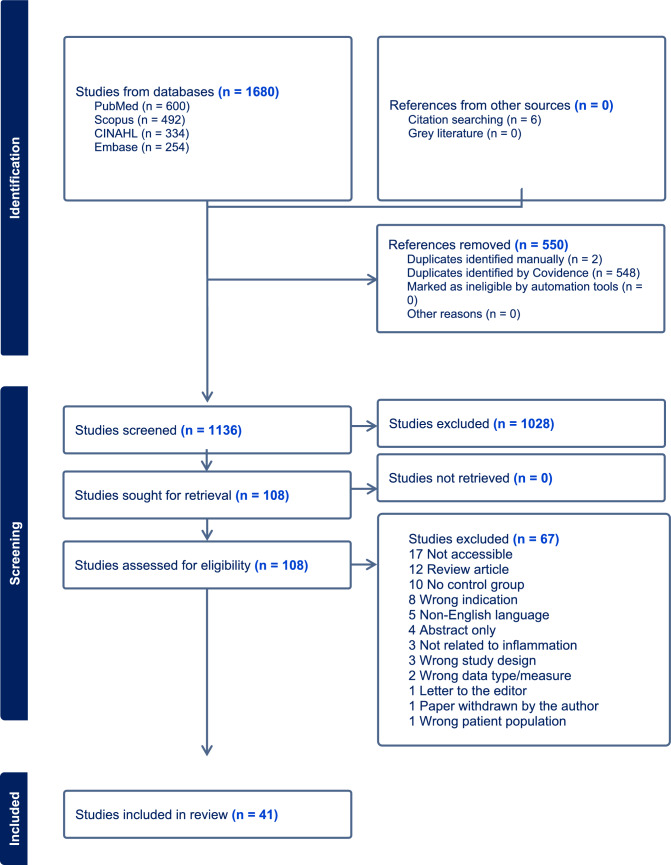
PRISMA flow diagram of the study selection process

A total of 1680 search results were identified during the active database and citation searches (PubMed 600, Scopus 492, CINAHL 334, EMBASE 254, and citation search 6) (Fig. 1). After the removal of 550 duplicate articles, 1136 articles underwent primary screening. Two reviewers were engaged in title/abstract screening independently according to the set inclusion and exclusion criteria. Following primary screening, 108 articles were retrieved for full-text assessment. Following full-text assessment, 67 articles were excluded because of the following reasons: abstract only (4), wrong study design (3), not accessible (17), review article (12), no control group (10), wrong indication (8), letter to the editor (1), non-English language (5), wrong patient population (1), article withdrawn by the author (1), wrong data type/measure (2), and not related to low-grade inflammation (3). Finally, 41 articles were included in the systematic review for data extraction and analysis (Fig. 1).

### Study Characteristics

Of the 41 included studies, all were published between 2002 and 2021; 19 were cross-sectional, 1 was comparative diagnostic, 3 were observational, 3 were retrospective, 3 were pilot, 5 were prospective cohort, 6 were case–control, and 1 was diagnostic accuracy (Table 1). From all included studies, there was a total of 1842 reported control cases and 2606 cases with IBS. Some studies included IBS subgroups; the total numbers of patients in different subgroups were 719 IBS-D, 224 IBS-C, 31 IBS-M, 11 IBS-A, 119 IBS-U, 38 NPIIBS (Non-post-infectious IBS), and 32 PIIBS (Post-infectious IBS). Only 3 studies included children (below 18 years) [17, 36, 37], with the remaining 35 studies including only adult participants [2, 3, 7, 11, 12, 14–16, 18–23, 25–28, 30–35, 60–70]. Thirty-four studies focused on determining direct or indirect inflammatory markers in patients with IBS and compared these markers with healthy controls [3, 7, 11, 12, 14–21, 25, 28, 31–33, 35–37, 60–63, 65, 67–75]. Ten studies focused on the changes in gut microbiota [2, 23, 25, 30, 34, 35, 64, 74]. Three studies focused on both detecting the presence of inflammatory markers and the changes in gut microbiota in line with low-grade inflammation [25, 35, 74] (Table 1).

**Table 1:** Demographic, clinical, and methodological features of studies included in the systematic review investigating immunological status and gut microbial dysbiosis in irritable bowel syndrome

Author	Year of publication	Country	Study type	Duration of study	Control group	IBS group	M/F ratio: control	M/F ratio: IBS	Age means: control	Age means: IBS	Focus of study
Summertona et al. [18]	2002	UK	Prospective	Not reported	28	7	Not reported	Not reported	Not reported	Not reported	Inflammatory marker
Costa et al. [20]	2003	Italy	Cross-sectional	Not reported	34	48	16:18	14/34	33.27 ± 21.26	43.53 ± 32.10	Inflammatory marker
Dolwani et al. [62]	2004	UK	Diagnostic accuracy	Not reported	26	24	18:08	20:04	39.33 ± 23.53	40.33 ± 40.19	Inflammatory marker
Reinders et al. [61]	2005	Sweden	First with a cross-sectional and the second with a longitudinal design	5 Years	28	39	16:12	11:28	Age range 18–81	Age range 20–47	Inflammatory marker
Dinan et al. [71]	2005	Ireland	Prospective		48	49	50/26	50/25	30.2 ± 13.5	34.6 ± 13.1	Inflammatory marker
Shulman et al. [36]	2008	USA	Retrospective	4.8 Years	46	76	7:39	12:65	8.5 ± 1.3	8.2 ± 1.4	Inflammatory marker
Clarke et al. [19]	2010	Ireland	Cross-sectional		26	41			39.04 ± 12.78	45 ± 11.47	Inflammatory marker
Del Valle-Pinero et al. [3]	2011	USA	Pilot	1.5 Years	12	12	6:6	5:7	28.00 ± 7.54	28.00 ± 7.54	Inflammatory marker
McKernan et al. [65]	2011	Ireland	Case control	Not reported	30	30 (11 IBS-A, 10 IBS-C, 9 IBS-D)	Not reported	Not reported	36.24 ± 1.835	40.9 ± 2.034	Inflammatory marker
Ohman et al. [32]	2012	Sweden	Cross-sectional	3 Months	30	74	10:20	22:52	39 ± 10	34 ± 16	Inflammatory marker
Rana et al. [33]	2012	India	Cross-sectional		62	63 IBS-D	32:30	37:26	43.5 + 18.7	42.6 + 19.5	Inflammatory marker
Fourie et al. [63]	2014	USA	Pilot	Not reported	31	(IBS-D = 5, IBS-C = 5, or IBS-M = 2) Total IBS 12	15:16	3:09	27.9 + 8.5	29.2 + 7	Inflammatory marker
Aktas et al. [60]	2014	Turkey	Retrospective	Not reported	44	40	18:26	13:27	39.3 ± 10.8	42.2 ± 15.2	Inflammatory marker
Melchior et al. [73]	2016	France	Prospective	Not reported	15	93	Not reported	Not reported	Not reported	41 ± 13.9	Inflammatory marker
Hod et al. [72]	2016	USA	Cross-sectional	2 Years	244	242 (101 IBS-D, IBS unspecified 89, IBS-C 39, IBS-M 13)	Not reported	Not reported	39.87 ± 15.04	38.78 ± 14.16	Inflammatory marker
Bennet et al. [75]	2016	Sweden	Observational	Not reported	58	173	22/36	54/119	27 (25–34)	30 (24–43)	Inflammatory marker
Patel et al. [11]	2017	India	Cross-sectional	1 Year	29	38 NPIIBS	2.75:1	3.8:1	38.3 ± 13.6	37.4 ± 13.7	Inflammatory marker
Choghakhori et al. [16]	2017	Iran	Case–control	1 Month	90	90	Not reported	Not reported	38.69 ± 9.21	37.66 ± 8.84	Inflammatory marker
Boyer et al. [15]	2017	France	Case–control	10 Months	20	11 (7 IBS-D, 4 IBS-C)	13:07	2:09	60.10 ± 16.52	IBS-C: 67.50 ± 13.65, IBS-D: 51.67 ± 37.66	Inflammatory marker
Du et al. [21]	2018	China	Cross-sectional	Not reported	46	112 IBS-D	22:44	53:59	43.1 ± 11.4	45.6 ± 12.7	Inflammatory marker
Linsalata et al. [28]	2018	Italy	Case–control	Not reported	20	39	7:13	6:33	39.7 ± 7.2	40.05 ± 12.2	Inflammatory marker
Popa et al. [68]	2018	Romania	Cross-sectional	4	37	39	13:24	16:23	39.00 ± 16.97	31 ± 8.47	Inflammatory marker
Miri et al. [67]	2018	Iran	Cross-sectional	Not reported	79	79	38:41	16/63	39.7 ± 18.9	42.5 ± 14.6	Bacteria causing low-grade inflammation
Chen et al. [2]	2018	China	Comparative diagnostic	2 Years	20	20 IBS-D	Not reported	Not reported	Not reported	Not reported	Low-grade inflammation by bacteria
Shukla et al. [35]	2018	India	Observational	Not reported	25	47	22.3	39.8	42.67 ± 37.73	40.33 ± 37.47	Inflammatory marker, low-grade inflammation by bacteria
Choi et al. [17]	2019	China	Prospective	3 Years	56	157	28:28	91:66	8.45 ± 3.39	9.48 ± 3.41	Inflammatory marker
Moraes et al. [31]	2020	Sweden	Cross-sectional	Not reported	21	40	9:12	14:26	33.33 ± 26.24	37.67 ± 32.30	Inflammatory marker
Aktas et al. [12]	2020	Turkey	Cross-sectional	2.7 Years	61	87	26:35	27:60	Median 38 (13)	Median 40 (26)	Inflammatory marker
Zhang et al. [70]	2020	China	Cross-sectional	Not reported	174	177 (IBS-D 77, IBS-C 27, PI-IBS 32, IBS-M 11 and IBS-U 30)	50:124	54:123	40.56 ± 14.28	39.12 ± 15.23	Inflammatory marker
Singh et al. [37]	2020	USA	Cross-sectional	Not reported	10	37	Not reported	Not reported		13.8 ± 2.2	Inflammatory marker
Berg et al. [14]	2020	Norway	Cross-sectional	3.4 Years	20	42	Not reported	Not reported	54.67 ± 27.92	Not reported	Inflammatory marker
Vaghari-Tabari et al. [69]	2020	Iran	Case–control	1.4 Years	50	50 (IBS-D 18, IBS-C 17, IBS-M 15)		27:23	36.00 ± 10.34	34.00 ± 9.93	Inflammatory marker
Jabbar et al. [26]	2020	Sweden	Prospective	5.10 Years	31	62	13/18	14:48	34.33 ± 27.98	36.67 ± 31.88	Low-grade inflammation by bacteria
Hong et al. [23]	2020	China	pilot explorative study		16	55 IBS-D	7/9	38:17	35 (10.94)	34.98 (10.34)	Low-grade inflammation by bacteria
Lee et al. [27]	2020	Korea	Cross-sectional	4 Years	12	7	Not reported	Not reported	Not reported	Not reported	Low-grade inflammation by bacteria
Liu et al. [64]	2020	China	Observational	Not reported	32	IBS-D 44	22/10	28/16	47.44 (11.630)	42.80 (9.002)	Low-grade inflammation by bacteria
Mei et al. [30]	2021	China	Case–control		30	30 IBS-D			40.9 ± 14.4	40.3 ± 14.7	Low-grade inflammation by bacteria
Ivashkin et al. [25]	2021	Russia	Cross-sectional	3 Months	10	31 (16 IBS-D; 15 IBS-C)	4:06	10:21	38.33 ± 7.31	33.17 ± 12.05	Inflammatory marker, low-grade inflammation by bacteria
Sciavilla et al. [34]	2021	Italy	Cross-sectional	Not reported	18	20	10:08	13:07	45.4 ± 5.8	46.4 ± 6.3	Low-grade inflammation by bacteria
Sun et al. [74]	2021	China	Cross-sectional	2 Years	66	162 IBS-D	Not reported	Not reported	Age range 18–65	Age range 18–65	Inflammatory marker, low-grade inflammation by bacteria
Güven et al. [7]	2022	Turkey	Retrospective	1.8 Years	107	107 IBS-C	49:58	40:67	45.3 ± 13.3	46.6 ± 15.2	Inflammatory marker

In this review, dysbiosis is defined based on the included studies as referring to reduced microbial diversity (e.g., lower alpha diversity), and/or altered microbial composition (e.g., imbalance between SCFA-producing and pro-inflammatory/pathogenic bacteria or fungi) [2, 23, 25–27, 34, 35, 64, 74]

*NPIIBS* non-post-infectious IBS, *PIIBS* post-infectious IBS, *IBS-C* constipation predominant IBS, *IBS-D* diarrhea predominant IBS, *IBS-U* unsubtyped/unclassified IBS, *IBS-M* mixed IBS-A: alternating IBS (alternating diarrhea and constipation) (often IBS-M and IBS-A are used interchangeably)

### Inflammatory Markers in IBS

Thirty-four studies detected many direct and indirect inflammatory markers, which are included in Table 2.

**Table 2:** Inflammatory markers with their abbreviations

Marker	Abbreviation	Marker	Abbreviation
IL-1 receptor antagonist	IL1-ra	Interferon-inducible protein 10	IP-10
Interleukin-1 beta	IL-1β	Monocyte chemotactic protein 1	MCP-1
Interleukin-2	IL-2	Macrophage inflammatory protein 1α	MIP-1α
Interleukin-4	IL-4	Macrophage inflammatory protein 1β	MIP-1β
Interleukin-5	IL-5	Platelet derived growth factor-BB	PDGF-BB
Interleukin-6	IL-6	Regulated upon activation, T cell expressed and secreted	RANTES
Interleukin-7	IL-7	Tumor necrosis factor	TNF
Interleukin-8	IL-8	Vascular endothelial growth factor	VEGF
Interleukin-9	IL-9	Malondialdehyde	MDA
Interleukin-10	IL-10	Total antioxidant capacity	TAC
Interleukin-12	IL-12	Macrophages	–
Interleukin-12 (p70)	IL-12 (p70)	T-lymphocytes	–
Interleukin-13	IL-13	Arachidonic acid	AA
Interleukin-15	IL-15	Prostaglandin E2	PGE2
Interleukin-17	IL-17	Leukotriene	LTB4
Tumor necrosis factor alpha	TNF-α	Intraepithelial lymphocytes	IELs
Soluble triggering receptor expressed on myeloid cells 1	sTREM-1	CD3+ T cells	–
TREM-1 protein and mRNA levels	TREM-1	Cortisol	–
Calprotectin	–	Claudin 3	CLD-3
Platelet	–	Claudin 5	CLD-5
Neutrophil	–	Chemokine receptor CXCR-3	CXCR-3
Neutrophil-to-lymphocyte ratio	NLR	Chemokine ligand CXCL-11	CXCL-11
Platelet-to-lymphocyte ratio	PLR	Chemokine (C-C motif) ligand 16	CCL-16
Systemic Immune-Inflammation Index	SII	Nitric oxide	NO
Interferon gamma	IFN-γ	Interferon-inducible protein 10	IP-10
White blood cell count	WBC	Granulocyte macrophage colony stimulating factor	GM-CSF
Hemoglobin	Hb	IL-1 receptor antagonist	IL1-ra
Platelet distribution width	PDW	Basic fibroblast growth factor	bFGF
Neutrophil–lymphocyte ratio	NLR	Granulocyte-colony stimulating factor	G-CSF
Mixed lymphocyte reaction	MLR	TH17 cell densities	–
Hematocrit	Htc	Eotaxin	–
Mean corpuscular volume	MCV	Red cell distribution width	RDW
Platelet count	PLT	Mean platelet volume	MPV
Platelet–lymphocyte ratio	PLR	Peroxiredoxin 1	PRDX1
High-sensitive C-reactive protein	hs-CRP	Eosinophils	–
Lipopolysaccharide	LPS	Mast cells	–
Toll-like receptor 4	TLR-4	hsa-miR-150	hsa-miR-150
Toll-like receptor 2	TLR-2	hsa-miR-342-3p	hsa-miR-342-3p

The most frequently assayed inflammatory markers were IL-6 (six studies) [11, 14, 21, 28, 33, 68], IL-10 (five studies) [11, 14, 16, 32, 33], IL-8 (four studies) [11, 14, 28, 65], TNF-α (four studies) [16, 21, 33, 70], and fecal calprotectin (seven studies) [17, 18, 20, 31, 36, 62, 66]. Three studies investigated eosinophils and mast cells as indirect markers of low-grade inflammation [15, 37, 67]. IL-2, IL-12, IL-1β, RDW, mast cell, MPV, and TLR-4 were detected by two studies each. Among these markers, IL-2, IL-12, IL-1β, and RDW are direct inflammatory markers, and the others are indirect inflammatory markers that do not cause low-grade inflammation directly but are actively engaged in the development of low-grade inflammation (Table 3).

**Table 3:** Levels of inflammatory markers between people with IBS and healthy control participants

Author	Sample type	Detection of markers	Result for IBS	Result for control	*p* value control vs. IBS
Patel et al. [11]	Blood	IL-2, IL-6, IL-8, IL-10	(For NPIIBS) IL-2: 96.3 (17.7), IL-6: 18.0 (9.8), IL-8: 92.5 (30.6), IL-10: 6 (0.0)	IL-2: 26.5 (4.9), IL-6: 8.0 (1.7), IL-8: 52.5 (10.6), IL-10: 17.5 (6.4)	IL-2: < 0.002, IL-6, 8, 10: NS (not significant)
Du et al. [21]	Serum	TNF-α, IL-1β, IL-6, sTREM-1, protein and mRNA level TREM-1	No difference in serum mucosa: TNF-α, IL-1β, and IL-6, and no difference in protein and mRNA level TREM-1 in biopsy. Significant increase of sTREM-1 in IBS-D. Median serum sTREM-1 levels 130.7 pg/mL	No difference in serum mucosa: TNF-α, IL-1β, and IL-6, and no difference in protein and mRNA level TREM-1 in biopsy	0.0052
Moraes et al. [31]	Serum	Calprotectin	Calprotectin, μg/g: 38.33 ± 53.83	Calprotectin, μg/g: 20.33 ± 12.72	Not reported
Ohman et al. [32]	Blood	IL-10, IL-12, IL-1β, TLR2, TLR4	Serum levels of IL-10 (*p* = 0.054), HLA-DR, CD86, CD40, CD80, TLR2 and TLR4 increased. LPS stimulated: IL-10: 17 870 ± 14 270, IL-12: 25 ± 33, IL-1β: 320 ± 323	IL-10: 21.285 ± 14.780, IL-12: 48 ± 74, IL-1β: 300 ± 390	TLR2 *p* = 0.0007. LPS stimulated: NS
Güven et al. [7]	Blood	Platelet, Neutrophil, NLR, PLR, and SII	[(× 10^3^/mm^3^), mean ± SD. Platelet 311.5 ± 66.9, Neutrophil 5466.4 ± 1360.7, Lymphocyte 2131.3 ± 590.7, SII 854.4 ± 376.1], NLR 2.7 ± 1.0, PLR 155.8 ± 51.5	Mean ± SD = Platelet 239.8 ± 50.4, Neutrophil 3946.5 ± 1040.6, Lymphocyte 2383.3 ± 630.3, SII 411.9 ± 145.0, NLR 1.7 ± 0.6, PLR 105.6 ± 29.0	Platelet < 0.001, Neutrophil < 0.001, Lymphocyte 0.003, SII < 0.001, NLR < 0.001, PLR < 0.001
Del Valle-Pinero et al. [3]	Blood	Chemokine (C-C motif) ligand 16 (CCL-16) gene and CCL-16 protein	Gene expression: (*n* = 12, Avg *C*_t_ = 30.67 ± 4.92). [IBS-C: (*n* = 4, Avg Ct = 25.59 ± 5.88]. IBS-D: (*n* = 8, Avg *C*_t_ = 33.16 ± 1.39). CCL-16 protein levels in plasma: (9.45 ± 3.25 ng/mL). [IBS-C (10.91 ± 2.58 ng/mL), IBS-D 6.06 ± 1.71 ng/mL]	Gene: *n* = 12, Avg *C*_t_ = 33.08 ± 1.39. Protein: 7.80 ± 4.20 ng/mL	Protein IBS vs. Control *p* = 0.34, protein IBS-C vs. IBS-D *p* = 0.018
Melchior et al. [73]	Feces	Fecal calprotectin	90.5 ± 180 mg/g	24.3 ± 16.1 mg/g	(*p* < 0.01)
Claudia et al. [61]	Rectal NO	NO	150 ppb (53–200 ppb)	f 45 ppb (34–64 ppb)	(*p* < 0.001)
Aktas et al. [12]	Venous blood	WBC, Hb, PDW, NLR, MLR, Htc, MCV, PLT, PLR	Median (IQR): WBC (k/mm^3^) = 7000 (2880), Hb (g/dL) = 14(2), PDW (%) = 16.3 (1), NLR (%) = 2.2 (1.1), MLR (%) = 0.25 (0.14). Mean ± SD: Htc (%) = 41.6 ± 3.2, MCV (fL) = 87 ± 4, PLT (k/mm3) = 259,000 ± 62,000, PLR (%) = 144 ± 50	Median (IQR): WBC (k/mm^3^) = 7590 (2450), Hb (g/dL) = 14.1 (1.4), PDW (%) = 15.4 (2.4), NLR (%) = 1.8 (0.7), MLR (%) = 0.2 (0.12). Mean ± SD: Htc (%) = 42 ± 3, MCV (fL) = 87 ± 4, PLT (k/mm^3^) = 259,000 ± 60,000, PLR (%) = 111 ± 32	Median (IQR): WBC (k/mm^3^) = 0.17, Hb (g/dL) = 0.20, PDW (%) = < 0.001, NLR (%) = < 0.001, MLR (%) = < 0.001. Mean ± SD: Htc (%) = 0.38, MCV (fL) = 0.95, PLT (k/mm^3^) = 0.99, PLR (%) = < 0.001
Hod et al. [72]	Peripheral blood	Serum hs-CRP (high-sensitive C-reactive protein)	1.80; IQR, 0.7 to 4.04 mg/L. hs-CRP was higher in IBS-D than IBS-C (4.98; IQR, 2.22 to 8.96 mg/L, IQR, 0.74 to 5.18 mg/L, vs. 1.10; IQR, 0.55 to 2.6 mg/L. Correlation of hs-CRP levels with symptoms severity (*r* = 0.169, *p* = 0.009), which was stronger for the IBS-D patients (*r* = 0.27, *p* = 0.006)	1.20; IQR, 0.5 to 2.97 mg/L	*p* < 0.006 IBS-C vs. IBS-D *p* = 0.0025
Linsalata et al. [28]	Peripheral venous blood	IL-6, IL-8, LPS, TLR-4	IL-6 4.3, IL-8 20, LPS 0.23, TLR-4 0.36	IL-6 2.5, IL-8 9, LPS 0.20, TLR-4 0.35	Among groups: IL-6: (*p* = 0.024), IL-8: (*p* < 0.0001), (LPS: *p* = 0.132, TLR-4: *p* = 0.832 = not significant). D-IBS vs. HC: IL-8: (*p* < 0.001)
Fourie et al. [63]	Blood	hsa-miR-150, hsa-miR-342-3p	hsa-miR-150 = 3988 ± 1195; has-miR-342-3p = 491 ± 152	hsa-miR-150 = 2509 ± 1135; hsa-miR-342-3p = 292 ± 87	FDR adjusted *p* ≤ 0.05 for both hsa-miR-150 and hsa-miR-342-3p
Popa et al. [68]	Peripheral venous blood	IL-6	IL-6 (pg/mL), 5.08 ± 0.95	IL-6 (pg/mL), 3.1 ± 0.62	*p* < 0.001
Aktas et al. [60]	Venous blood	RDW (red cell distribution width), MPV (mean platelet volume)	Mean RDW: 0.165 ± 0.0037. MPV: 8.27 ± 1.07	Mean RDW: 0.160 ± 0.006. MPV: 7.80 ± 1.01	RDW: *p* < 0.001, MPV: *p* = 0.046
Zhang et al. [70]	Blood	TNF-α and PRDX1	TNF-α = 22.40, IQR 9.30–24.50 pg/mL, PRDX1 = 9.8, IQR 7.2–11.90 ng/mL	TNF-α = 8.30, IQR 7.50–9.30 pg/mL, PRDX1 = 3.4, IQR 2.9–4.20 ng/mL	TNF-α = *p* < 0.001, PRDX1 = *p* < 0.001
Shulman et al. [36]	Feces	Fecal calprotectin	(µg/g Stool) = 65.5 ± 75.4	(µg/g Stool) = 43.2 ± 39.4	*p* < 0.01
Singh et al. [37]	Biopsy	Eosinophil, mast cell, and TH17 cell densities	Rectosigmoid: Eosinophil = 19.0 ± 11.4, Mast cell = 19.8 ± 6.4, TH17 cell = 1.2 ± 0.5. Descending: Eosinophil = 26.8 ± 15.1, Mast cell = 21.5 ± 8.0, TH17 cell = 1.2 ± 0.6	Rectosigmoid: Eosinophil = 10.1 ± 6.0, Mast cell = 10.7 ± 6.3, TH17 cell = 0.5 ± 0.5. Descending: Eosinophil = 15.2 ± 4.0, Mast cell = 8.7 ± 2.9, TH17 cell = 0.4 ± 0.5	Rectosigmoid: Eosinophil = 0.022, Mast cell = < 0.001, TH17 cell = 0.001. Descending: Eosinophil = < 0.001, Mast cell = < 0.001, TH17 cell = 0.001
Rana et al. [33]	Peripheral venous blood	IL-6, TNF-α and IL-10	IL-6 = (32.2 ± 12.01 pg/mL), TNF-α = (16.3 ± 5.2), IL-10 = 5.75 ± 2.1	IL-6 = (7.48 ± 2.55 pg/mL), TNF-α = (7.94 ± 2.19 pg/mL), IL-10 = 5.84 ± 1.9	IL-6 = (*p* < 0.001), TNF-α = (*p* < 0.05)
Berg et al. [14]		IL-1b, IL-1 receptor antagonist (IL1-ra), IL-2, IL-4, IL-5, IL-6, IL-7, IL-8, IL-9, IL-10, IL-12 (p70), IL-13, IL-15, IL-17, eotaxin, bFGF, G-CSF, GM-CSF, (IFN)-c, IP-10, MCP-1, MIP-1α, MIP-1β, PDGF-BB, RANTES, TNF and VEGF	IFNc 18 (16–20), IL-1β 6.7 (5.6–7.9), IL-2 0.64 (0.58–0.71), IL-4 0.91 (0.79–1.04), IL-9 8.2 (7.2–9.2), IL-15 2.3 (2.1–2.6), IL-17 3.2 (2.6–3.7), TNF 160 (143–178), eotaxin 6.7 (5.6–7.9), RANTES 2011 (1378–2934), bFGF 674 (596–761), GM-CSF 4.4 (3.8–5.1), PDGFBB 11.7 (9.9–13.7), IP-10 = 590 (437–798), MCP-1a = 75 (66–86), MIP-1α = 3.3 (2.6–4.2), MIP-1β = 74 (62–89)	IFNc 14 (12–17), IL-1β 4.1 (3.1–5.5), IL-2 0.39 (0.35–0.44), IL-4 0.71 (0.62–0.82), IL-9 6.5 (5.7–7.5), IL-15 1.7 (1.5–1.8), IL-17 2.0 (1.6–2.4), TNF 130 (115–147), eotaxin 4.1 (3.1–5.5), RANTES 891 (545–1454), bFGF 446 (384–518),GM-CSF 2.4 (1.7–3.1), PDGFBB 8.2 (6.1–11.1), IP-10 = 401 (301–533), MCP-1a = 71 (60–85), MIP-1α = 3.0 (2.3–4.0), MIP-1β = 64 (53–77)	IFNc 0.10, IL-1β 0.004, IL-2 < 0.0005, IL-4 0.032, IL-9 0.040, IL-15 < 0.0005, IL-17 0.001,TNF 0.025, eotaxin 0.004, RANTES 0.016, bFGF < 0.0005, GM-CSF < 0.0005, PDGFBB 0.032
Vaghari-Tabari et al. [69]	Blood	RDW (red cell distribution width), MPV (mean platelet volume)	RDW % ± SD = 13.09 ± 1.70, MPV fL ± SD = 9.24 ± 0.80	RDW % ± SD = 12.56 ± 0.69, MPV fL ± SD = 10.11 ± 0.74	RDW *p* 0.047, MPV *p* 0.001
Choghakhori et al. [16]		Tumor necrosis factor (TNF)-α, interleukin (IL)-17, interleukin (IL)-10, malondialdehyde (MDA), and total antioxidant capacity (TAC)	Increase: TNF-α (14.7 ± 5.27 pg/mL), IL-17 (8.46 ± 3.32 pg/mL), MDA (3.85 ± 1.53 ng/mL). Decrease: IL-10 (7.02 ± 3.07 pg/mL), TAC (736.87 ± 151.74 μmol/L)	TNF-α (10.04 ± 5.75 pg/mL), IL-17 (5.63 ± 3.15 pg/mL), MDA (2.36 ± 1.54 ng/mL), IL-10 ( 9 ± 3.23 pg/mL), TAC (941.11 ± 166.22 μmol/L)	TNF-α (*p* < 0.001), IL-17 (*p* < 0.001), MDA (*p* < 0.001), IL-10 (*p* < 0.001), TAC (*p* < 0.001)
Choi et al. [17]	Feces	Fecal calprotectin	Mean of FC (µg/g): 83.50 ± 164.74	Mean of FC (µg/g): 17.77 ± 10.92	*p* < 0.001
Boyer et al. [15]	Colonic biopsy	Macrophages, mast cells, eosinophils, and T-lymphocytes	MP's elevated in the left colon (99.5 [86.8–123]. MC decreased significantly	Mp's 82.8 [72–97.5]. MC was higher than the IBS group	Mp's *p* = 0.04. MC *p* = 0.04
Christopher et al. [18]	Feces	Fecal calprotectin	Mean 11, SD 12.65, Median 6.1 mg/L, range 1.9–32.0	Mean 5.1, SD 3.90, Median 4.5, range 0.9–15.5	Not reported
Costa et al. [20]	Feces	Fecal calprotectin	Normal median value but increased than HC (22 µg/g, 95% CI 9–35 µg/g)	Normal median value HC (11 µg/g, 95% CI 3–18 µg/g)	Not reported
Dolwani et al. [62]	Feces	Fecal calprotectin	(µg/g) 36.23 ± 66.43	(µg/g) 18.33 ± 32.16	*p* = 0.04
Clarke et al. [19]	Plasma	Arachidonic acid (AA), eicosanoids—PGE2, LTB4	AA = 8.52 ± 0.26 g/100 g, PGE2 = 1490 ± 142.3 pg/mL, LTB4 = 332.2 ± 31.33 pg/mL	AA = 7.53 ± 0.37 g/100 g, PGE2 = 934.2 ± 145.7 pg/mL, LTB4 = 226 ± 36.70 pg/mL	AA = *p* 0.029, PGE2 = *p* 0.01, LTB4 = *p* 0.03
Miri et al. [67]	Biopsy	Intraepithelial lymphocytes (IELs), eosinophils, mast cells, and CD3 + T cells	IELS = 32.8 ± 11.8, CD3+ T = 23.1 ± 7.9, eosinophil 5.4 ± 2.9, mast cells = 7.6 ± 3.1	IELS = 28.6 ± 12.9, CD3+ T = 20.2 ± 8.1, eosinophil 4.5 ± 3.2, mast cells = 6.6 ± 3.0	IELS = 0.034, CD3+ T = 0.024, eosinophil = 0.066, mast cells = 0.041
McKernan et al. [65]	Plasma	Cytokines and cortisol	Cortisol 107,000 ± 12,340, IL-6 2.875 ± 0.3350, IL-8 9.397 ± 0.7667	Cortisol 78,220 ± 7590, IL-6 1.581 ± 0.1462, IL-8 7.474 ± 0.3745	Cortisol *p* 0.0488, IL-6 *p* 0.0008, IL-8 *p* 0.0282
Ivashkin et al. [25]	Mucosal biopsy,	CLD-3, CLD-5, IL-2, IL-10, and TNF-α	TNF-α = duodenum 3.5 ± 0.5, ileum 3.6 ± 0.5, cecum (5.4 ± 0.5, sigmoid colon 5.6 ± 0.5). IL-2 = duodenum 3.4 ± 0.6, ileum 3.7 ± 0.6, cecum 5.1 ± 0.8, sigmoid colon 5.3 ± 0.8. IL-10 = duodenum 1.9 ± 0.3, ileum 1.8 ± 0.4, cecum 1.7 ± 0.4, sigmoid colon 1.7 ± 0.5. CDL-3 = (duodenum 2.3 ± 0.7, ileum 2.3 ± 0.6, cecum 2.3 ± 0.6, sigmoid colon 1.1 ± 0.5. CDL-5 = duodenum 2.2 ± 0.5, ileum 2.1 ± 0.5, cecum 2.2 ± 0.5, sigmoid colon 0.8 ± 0.3	TNF-α = duodenum 1.1 ± 0.3, ileum 1.1 ± 0.3, cecum 1.0 ± 0.2, sigmoid colon 1.2 ± 0.3. IL-2 = duodenum 1.50 ± 0.51, ileum 1.5 ± 0.5, cecum 1.4 ± 0.5, sigmoid colon 1.3 ± 0.5. IL-10 = duodenum 3.9 ± 0.5, ileum 4.0 ± 0.0, cecum 4.5 ± 0.8, sigmoid colon 4.5 ± 0.8. CDL-3 = duodenum 5.6 ± 0.8, ileum 5.8 ± 0.6, cecum 5.7 ± 0.7, sigmoid colon 5.6 ± 0.8. CDL-5 = duodenum 5.9 ± 0.4, ileum 5.7 ± 0.7, cecum 6.0 ± 0.0, sigmoid colon 5.9 ± 0.5	TNF-α = *p* < 0.0001 for all localizations. IL-2 = *p* < 0.0001 for all localizations. IL-10 = *p* < 0.0001 for all localizations. CDL-3 = < 0.0001 for all localizations. CDL-5 *p* < 0.0001 for all localizations
Shukla et al. [35]	Biopsy	mRNA levels of TLR-4, TLR-5, IL-6, CXCL-11, CXCR-3, IL-10	mRNA levels of TLR-4 median 0.24 (0.0–26.6) ↑ 4.2-folds, TLR-5 0.77 (0.0–6.5) ↑ 6.6 folds. mRNA levels of IL-6 0.90 (0.0–5.0), CXCL-11–2.80 (0.0–98.8), CXCR-3 1.80 (0.0–24.8). Upregulated expression of IL-6, CXCL 11, CXCR 3. IL-10 downregulated	mRNA levels of TLR-4 median 0.03 (0.0–13.1), TLR-5 0.03 (0.0–1.5). mRNA levels of IL-6 0.10 (0.0–2.8), CXCL-11 0.10 (0.0–15.4), CXCR-3 0.00 (0.0–25.2)	Copy number of *Lactobacillus* (*p* = 0.045) and *Bifidobacterium* (*p* = 0.011) showed correlation with IL-10 in IBS-C, while Gram-positive (*p* = 0.031) and Gram-negative bacteria (*p* = 0.010) showed correlation with CXCL-11 in IBS-D patients
Dinan et al. [71]	Blood	IL-6, sIL-6R, IL-8, IL-10, TNF-α	IL-6 (mean ± SEM): in IBS patients 2.45 ± 0.19 pg/mL. sIL-6R: in IBS patients 92,760 ± 3077 pg/mL. IL-10: in IBS patients 4.65 ± 0.94 pg/mL. IL-8: in IBS patients 4.18 ± 0.30 pg/mL. TNF-α = no difference	IL-6 (mean ± SEM): in controls 1.07 ± 0.10 pg/mL. sIL-6R: in controls 54,960 ± 2616 pg/mL. IL-10: in controls 5.95 ± 0.94 pg/mL. IL-8: in controls 1.11 ± 0.11 pg/mL. TNF-α = no difference	IL-6: *p* < 0.001, sIL-6R: *p* < 0.001, IL-10: not significant, IL-8: *p* < 0.001, TNF-α: not significant
Sun et al. [74]	Plasma	IL-10, IL-12, MCP-1	Significantly higher IL-12 in IBS	Significantly lower IL-12 in control	(*p* = 0.037)
Bennet et al. [75]	Serum	IL-5, IL-6, IL-8, IL-10, IL-12p70, IL-13, IL-17A, IFN-γ, TNF	IL-5 = 0.3 (0.15–0.5), IL-6 = 0.4 (0.28–0.68), IL-8 = 11.3 (7.51–14.41), IL-10 = 0.25 (0.17–0.37), IL-12p70 = 0.16 (0.09–0.24), IL-13 = 1.19 (0.78–1.72), IL-17A = 0.9 (0.6–1.5), IFN-γ = 8.0 (5.1–12.1), TNF = 2.3 (1.8–2.7)	IL-5 = 0.2 (0.12–0.4), IL-6 = 0.34 (0.24–0.54), IL-8 = 9.1 (6.49–13.15), IL-10 = 0.27 (0.14–0.38), IL-12p70 = 0.15 (0.1–0.23), IL-13 = 1.01 (0.85–1.58), IL-17A = 0.8 (0.5–1.3), IFN-γ = 10.9 (6–15.3), TNF = 2.3 (1.6–2.6)	*p* values IL-5 = 0.56, IL-6 = 0.06, IL-8 = 0.12, IL-10 = 0.87, IL-12p70 = 0.64, IL-13 = 0.47, IL-17A = 0.24, IFN-γ = 0.07, TNF = 0.28

All studies showed differences in inflammatory responses between the control group and IBS group except one study where there was no difference in TNF-α, IL-1β, and IL-6, protein, and mRNA level of TREM-1 [21]. Several inflammatory markers were found to be altered considerably in people with IBS compared to healthy controls. IL-10 and lymphocytes were consistently decreased, whereas other markers (for example, IL-2, IL-6, IL-8, TNF-α, and fecal calprotectin) were elevated (*p* < 0.001) across multiple investigations [7, 12, 14, 16, 17, 20, 25, 28, 32, 33, 35, 37, 60, 61, 68–70]. However, some markers exhibited inconsistent outcomes throughout different studies. One study reported an increase in IL-10, while the majority found it to be decreased in people with IBS. Similarly, MPV, TNF-α, and IL-6 were generally increased, but some studies showed no difference between IBS and control participants [7, 11, 14, 16, 21, 32, 71]. These trends are summarized in Table 4, where the overall direction of change in inflammatory markers is presented.

**Table 4:** Summary of the comparison of inflammatory markers among IBS and control participants

Increase in inflammatory markers in IBS patients	References	Decrease in inflammatory markers in IBS patients	References	No changes in inflammatory markers between IBS and control participants	References
IL-1β, IL-2, IL-4, IL-5, IL-6, IL-8, IL-9, IL-10, IL-12p70, IL-12, IL-13, IL-17, sIL-6R, TNF-α, SII, NLR, PLR, PLT, TLR2, TLR4, PDW, RDW, NO, LPS, MPV, Hb, MP, PRDX1, TH-17, AA, IELS, PGE2, LTB4, GM-CSF, MDA, IFNc, IFN-γ, RANTES, bFGF, PDGF-BB, CD+ 3T, hs-CRP, has-miR-150, has-miR-342-3p, CCL-16, cortisol, eotaxin, mast cell, eosinophil, neutrophil, calprotectin, IL-10, MCP-1, MIP-1αβ	[3, 7, 11, 12, 14–17, 25, 31–33, 36, 37, 60, 61, 63, 65–68, 70–72, 74–76]	IL-10, IL-12, WBC, Htc, MC, TAC, CLD-3, lymphocyte, MPV, platelet	[7, 12, 15, 16, 32, 33, 69, 71]	TNF-α, IL-1β, IL-6, MCV, mRNA level TREM-1,	[21, 71]

**Fig. 2:**
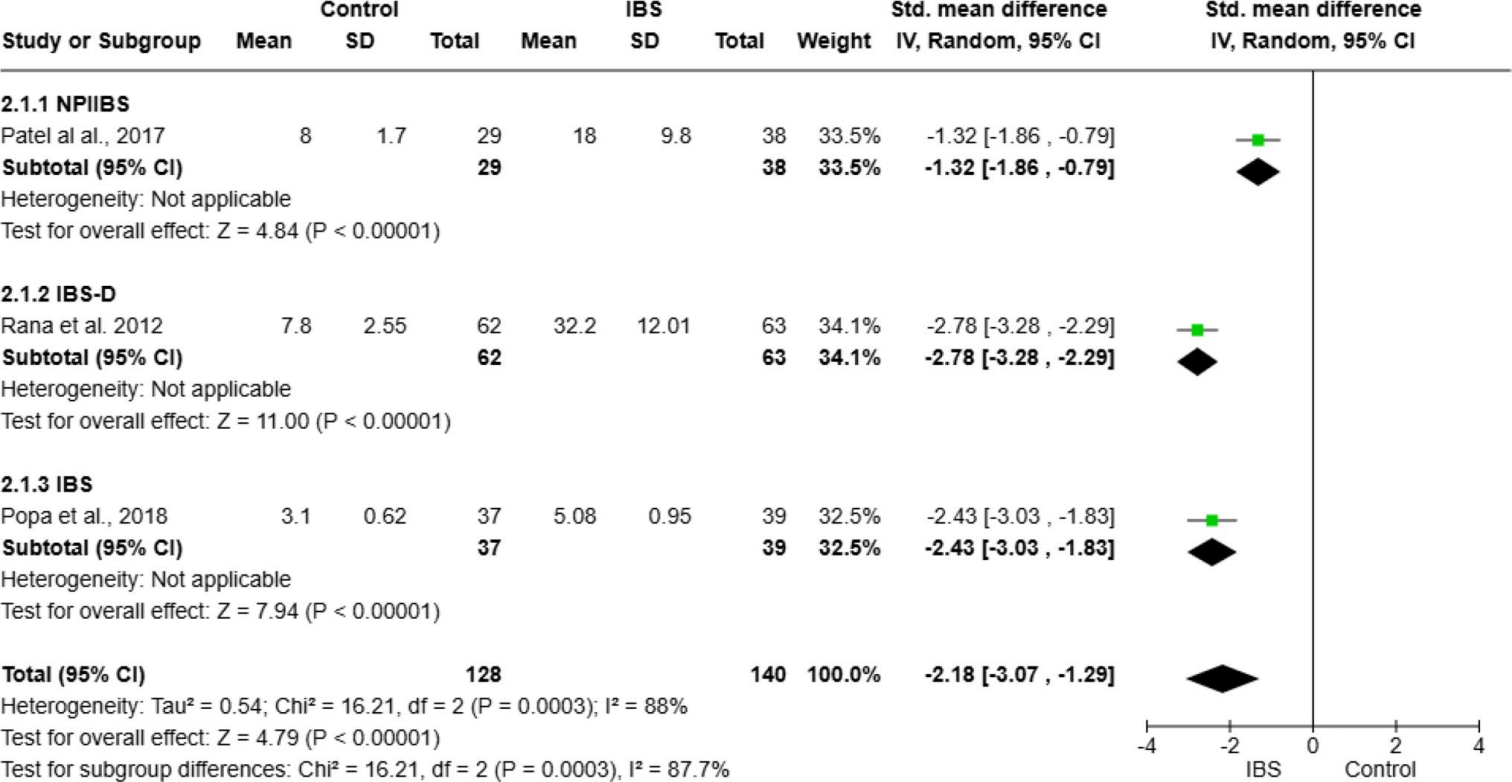
Meta-analysis of IL-6 between people with IBS and healthy control participants

Meta-analysis was performed on IL-6 (data from three studies [11, 33, 68], Fig. 2), fecal calprotectin (data from six studies [2, 18, 31, 36, 62, 66], Fig. 4), TNF-α (data from four studies [16, 25, 33, 70], Fig. 5), platelet–lymphocyte ratio (PLR, data from two studies [7, 12], Fig. 7), and IL-10 (data from three studies [16, 25, 32], Fig. 8). The meta-analysis showed that IL-6, TNF-α, PLR, and fecal calprotectin were greater in people with IBS than in healthy control participants (*p* values all > 0.05). On the contrary, IL-10 was lower in those with IBS compared with healthy controls (*p* < 0.00001).

This forest plot shows the meta-analysis of serum IL-6 levels between people with IBS and healthy controls. Subgroup analyses include NPIIBS (Non-post-infectious IBS), IBS-D, and overall IBS (not recruited as specific types of IBS). Pooled standardized mean differences (SMDs) indicate higher IL-6 levels in those with IBS (SMD − 2.18 [− 3.07, 1.29], *p* < 0.00001) with high heterogeneity (*I*^2^ = 87.7%).

This forest plot shows the sensitivity analysis of the meta-analysis of serum IL-6 levels between patients with IBS and healthy controls. To reduce the heterogeneity, the NPIIBS study was excluded from the meta-analysis, which resulted in low heterogeneity (*I*^2^ = 0%). Sensitivity analysis shows that removing any study does not significantly change the overall result, confirming that the findings are stable. Excluding one study can reduce heterogeneity to 0%, indicating that the study was the primary source of variability.

This forest plot shows the meta-analysis of fecal calprotectin levels between people with IBS and healthy controls. Subgroup analyses include the ages of participants —adults, children, and ages not mentioned. Pooled standardized mean differences (SMDs) indicate significantly higher calprotectin levels in those with IBS (SMD − 0.42 [− 0.61, − 0.24], *p* < 0.00001) with no heterogeneity (*I*^2^ = 0%).

This forest plot shows the meta-analysis of serum TNF-α levels between people with IBS and healthy controls. Subgroup analyses include the types of studies—cross-sectional and case–control. Pooled standardized mean differences (SMDs) indicate significantly higher TNF-α levels in those with IBS (SMD − 1.98 [− 2.79, − 1.17], *p* < 0.00001) with high heterogeneity (*I*^2^ = 86.0%).

This forest plot shows the sensitivity analysis of the meta-analysis of serum TNF-α levels between people with IBS and healthy controls. To reduce the heterogeneity, one study was excluded from the cross-sectional study subgroup of the meta-analysis, which resulted in low heterogeneity for subgroup difference (*I*^2^ = 29.4%). Sensitivity analysis shows that removing any study did not significantly change the overall result, confirming that the findings are stable. Excluding one study can reduce heterogeneity, indicating the study was the primary source of variability.

This forest plot shows the meta-analysis of serum PLR levels between people with IBS and healthy controls. Pooled standardized mean differences (SMDs) indicate significantly higher PLR levels in those with IBS (SMD − 0.98 [− 1.42, − 0.55], *p* < 0.00001) with substantial heterogeneity (*I*^2^ = 73%).

This forest plot shows the meta-analysis of serum IL-10 levels between people with IBS and healthy controls. Pooled standardized mean differences (SMDs) indicate significantly lower IL-10 levels in those with IBS (SMD 2.00 [1.69, 2.31], *p* < 0.00001) with no heterogeneity (*I*^2^ = 0%).

**Fig. 3:**
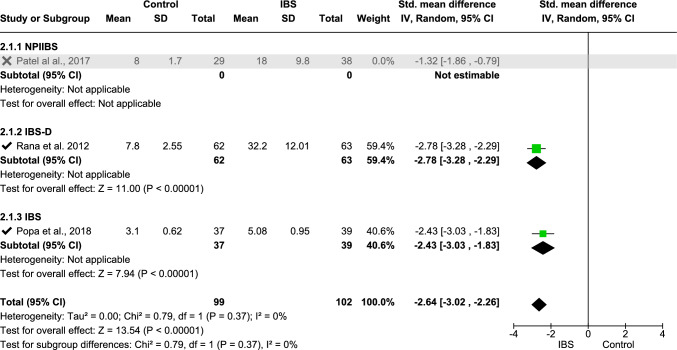
Sensitivity analysis for IL-6 between people with IBS and healthy control participants

**Fig. 4:**
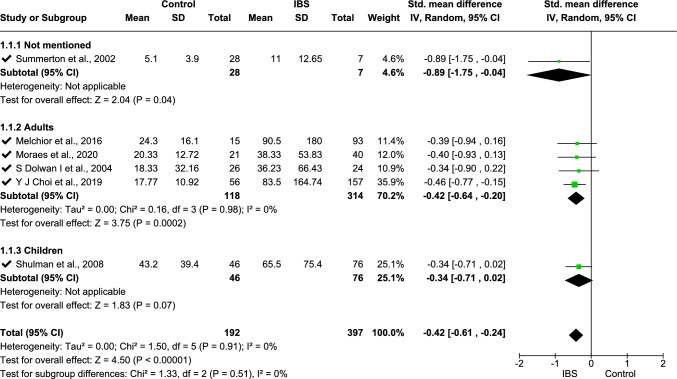
Meta-analysis on fecal calprotectin between people with IBS and healthy control participants

For IL-6, further analysis on three IBS subgroups was conducted: NPIIBS (non-post-infectious IBS), IBS (general IBS without any mention of specific IBS type), and IBS-D (Fig. 2). Heterogeneity was found *I*^2^ = 88% with overall test effect *Z* = 4.79 (*p* < 0.00001) and test for subgroup differences *I*^2^ = 87.7%. After sensitivity analysis, one study was withdrawn, which resulted in a subsequent *I*^2^ = 0% heterogeneity for both overall analysis and subgroup analysis (Fig. 3) with a standardized mean difference of − 2.64 (− 3.02, − 1.26), *p* < 0.00001. A moderate level of heterogeneity *I*^2^ = 73% was found in the meta-analysis of PLR (Fig. 4) with a standardized mean difference of − 0.98 (− 1.42, − 0.55), *p* < 00001.

**Fig. 5:**
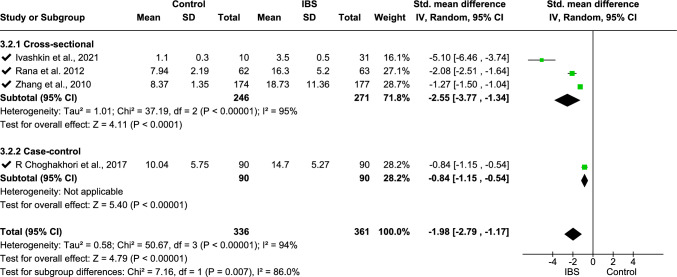
Meta-analysis on TNF-α between people with IBS and healthy control participants

**Fig. 6:**
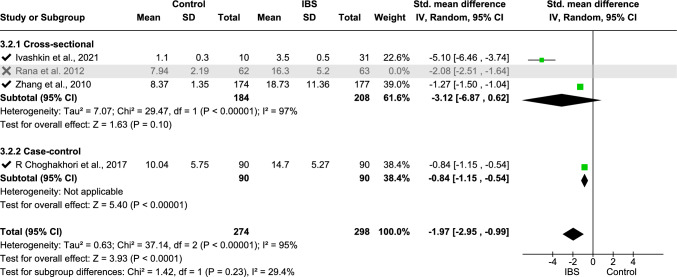
Sensitivity analysis on TNF-α sensitivity between individuals with IBS and healthy control participants

**Fig. 7:**
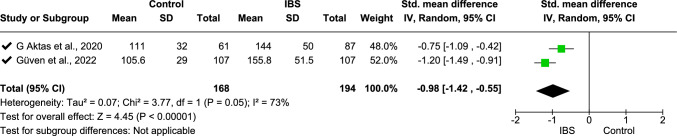
Meta-analysis on PLR (Platelet lymphocyte ratio) between individuals with IBS and healthy control participants

**Fig. 8:**
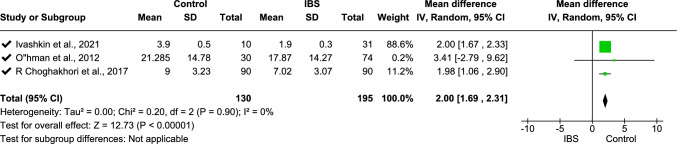
Meta-analysis on IL-10 between individuals with IBS and healthy control participants

For fecal calprotectin, a subgroup analysis was undertaken using three age groups (children, adults, and age not mentioned): this showed *I*^2^ = 0% heterogeneity (Fig. 5) and standardized mean difference − 0.42 (− 0.61, − 0.24), *p* < 0.00001. The overall heterogeneity of TNF-α analysis using two subgroups regarding study type (case–control study, cross-sectional study) was 94%, with 86% subgroup difference in heterogeneity using the standard mean-random effect model (Fig. 6). Following sensitivity analysis, one study [33] was withdrawn from the analysis, which reduced the subgroup difference heterogeneity to 29.4%, whereas overall heterogeneity was increased (95%), and the standardized mean difference was − 1.97 (− 2.95, − 0.99), *p* < 0001 (Fig. 7). For IL-10, mean difference with random effect was conducted with *I*^2^ = 0 heterogeneity and mean difference 2.00 (1.69, 2.31), *p* < 0.00001 (Fig. 8).

### Changes in Gut Microbiota

Nine studies included data on changes in gut bacteria [2, 23, 25–27, 30, 34, 35, 64]. The most frequently reported phyla were as follows: Firmicutes, Fusobacteria, Actinobacteria, Proteobacteria, Bacteroidetes, and Verrucomicrobia. Several families were also highlighted, including Acidaminococcaceae, Sutterellaceae, Desulfovibrionaceae, Enterococcaceae, Leuconostocaceae, Clostridiaceae, Peptostreptococcaceae, and Lachnospiraceae.

At the class level, Gammaproteobacteria and Clostridia were frequently reported. Additionally, studies identified changes in several unclassified genera, such as *Eggerthella, Lactococcus, Turicibacter, Fusobacterium, Coprococcus, Lachnobacterium, Faecalibacterium, Ruminococcus*, and *Akkermansia*.

Shifts in the Firmicutes/Bacteroidetes (*F*/*B*) ratio were also reported, along with differences in the abundance of genera like *Alloprevotella, Enterobacteriaceae, Parabacteroides, Flavonifractor, Phascolarctobacterium,* and *Parasutterella*.

Other microbial features studied included the copy number of *Lactobacillus* and *Bifidobacterium*, levels of IgA^+^ cells, and the presence of *Escherichia–Shigella, Granulicatella*, and *Haemophilus*. Some studies also examined *Brachyspira*-derived peptides.

While considering fungi, changes were noted in the phylum Zygomycota, and genera including *Mycosphaerella, Aspergillus, Sporidiobolus,* and *Pandora* (Table 4).

According to Table 5, at the phylum level, Firmicutes, Fusobacteria, and Actinobacteria were decreased, whereas Proteobacteria and Bacteroidetes were increased in those with IBS [23, 25, 27, 30]. Furthermore, at the class level, *Gamma proteobacteria* were more abundant and *Clostridia* less abundant in people with IBS [23]. At the genus level, *Alloprevotella* and *Fusobacterium* were reduced, while *Enterobacteriaceae* were considerably higher [30].

**Table 5:** Differences in gut bacteria between individuals with IBS and healthy control participants

Author	Sample type	Microbiota analytic technique	Sample size	IBS subtype	Detection of bacteria	Result for IBS	Result for control	*p* value control vs. IBS
Mei et al. [30]	Feces	16S rDNA gene sequencing	30 IBS-D, 30 healthy	IBS-D	Phylum: Firmicutes, Fusobacteria, Actinobacteria, Proteobacteria. Genus: *Alloprevotella, Fusobacterium, Enterobacteriaceae*	Phylum: decreased = Firmicutes, Fusobacteria, Actinobacteria; increased = Proteobacteria. Genus: decreased = *Alloprevotella*, *Fusobacterium*; increased = *Enterobacteriaceae*		Phylum: decreased = Firmicutes (*p* < 0.05), Fusobacteria (*p* < 0.01), Actinobacteria (*p* < 0.01); increased = Proteobacteria y (*p* < 0.01). Genus: decreased = *Alloprevotella* (*p* < 0.01), *Fusobacterium* (*p* < 0.01); increased = *Enterobacteriaceae* (*p* < 0.01)
Sciavilla et al. [34]	Feces	16S rDNA gene sequencing	20 IBS, 18 control	Not mentioned	*Actinomyces*, *Rothia, Gemella, Streptococcus, Enterococcus, Granulicatella, Fusobacterium, Escherichia/Shigella, Veillonella, and Clostridium* cluster XI. cultivable fungi, fungal species richness. *Candida albicans, Candida glabrata, Galactomyces*	Increased *Actinomyces*, *Rothia, Gemella, Streptococcus, Enterococcus, Granulicatella, Fusobacterium, Escherichia/Shigella, Veillonella, and Clostridium* cluster XI. Decreased Anaerostipes, Fusicatenibacter, Odoribacter, Oscillibacter, Roseburia, and Faecalibacterium	Decreased *Anaerostipes, Fusicatenibacter, Odoribacter, Oscillibacter, Roseburia, and Faecalibacterium*	Cultivable fungi: *p* = 0.028, fungal sp richness: *p* = 0.07, growth of *C. albicans*: (*p* < 0.05), hyphae *p* < 0.01
						Increased cultivable fungi, fungal species richness. *C. albicans, Candida glabrata, Galactomyces*. Lower growth of *C. albicans* with hyphae production	Increased *Anaerostipes*, Fusi*catenibacter, Odoribacter, Oscillibacter, Roseburia*, and *Faecalibacterium*. Decreased cultivable fungi and fungal species richness. *Candida. albicans, Candida parapsilosis, Aspergillus, Rhodotorula*. Higher growth of *C. albicans* and no hyphae production. *Penicillium*, and *Pichia* genera, fungal sp—*Torulaspora delbrueckii* and *Starmerella bacillaris*	
Ivashkin et al. [25]	Feces (microbiome)	16S rRNA gene sequencing	31 IBS (16 IBS-D, 15 IBS-C), 10 control	IBS-C, IBS-D	Qualitative and quantitative composition of the intestinal microbiota	Bacteria phylum level: Increased Bacteroidetes 19.9 [10.3; 40.2], Decreased Firmicutes 65.0 [53.3; 82.8]	Bacteria phylum level: Bacteroidetes 3.8 [0.0; 22.9], Firmicutes 85.0 [72.3; 96.1]	*p* < 0.05 for both phyla
Jabbar et al. [26]	Sigmoid colon biopsy	16S rRNA gene sequencing, qPCR, immunohistochemistry	62 IBS, 31 healthy	IBS-C, IBS-D, IBS-M, IBS-U	*Brachyspira*-derived peptides	Positive in 19/32 IBS	Absent in HS 0/31	(*p* < 0.001)
Hong et al. [23]	Feces	16s rRNA and ITS2 high-throughput sequencing	55 IBS-D, 16 healthy control	IBS-D	Phyla level: Proteobacteria, Firmicutes; Class level: Gammaproteobacteria, Clostridia, Eggerthella, Lactococcus, Turicibacter, Fusobacterium, Coprococcus, Lachnobacterium, Faecalibacterium, Ruminococcus, Akkermansia, Firmicutes/Bacteroidetes (F/B) ratio; 4 fungal markers: Mycosphaerella, Aspergillus, Sporidiobolus, and Pandora; fungal bacterial relation	Phyla level: Increased Proteobacteria, decreased Firmicutes. Class level: Increased Gammaproteobacteria, decreased Clostridia. Increased Eggerthella, Lactococcus, Turicibacter, Fusobacterium, decreased Coprococcus, Lachnobacterium, Faecalibacterium, Ruminococcus, Akkermansia. Increased Firmicutes/Bacteroidetes (*F*/*B*) ratio. Differed fecal fungal structure decreased, phyla Zygomycota decreased, 4 fungal markers: Mycosphaerella, Aspergillus, Sporidiobolus, and Pandora decreased, weak fungal bacterial relation	Phyla level: Decreased Proteobacteria, increased Firmicutes. Class level: Decreased Gammaproteobacteria, increased Clostridia. Decreased Eggerthella, Lactococcus, Turicibacter, Fusobacterium, increased Coprococcus, Lachnobacterium, Faecalibacterium, Ruminococcus, Akkermansia. Decreased Firmicutes/Bacteroidetes (*F*/*B*) ratio. Differed fecal fungal structure increased, and phyla Zygomycota increased. Strong fungal bacterial relation	F/B (*p* = 0.087). Fecal fungal structure *p* = 0.001. Phyla Zygomycota *p* < 0.001
Lee et al. [27]	feces	16S rRNA metagenomic sequencing	7 IBS-D, 12 control	IBS-D	Firmicutes, Bacteroidetes, Verrucomicrobia, Proteobacteria, Fusobacteria, Actinobacteria, Firmicutes/Bacteroidetes ratio. Acidaminococcaceae, Sutterellaceae, Desulfovibrionaceae, Enterococcaceae, Leuconostocaceae, Clostridiaceae, Peptostreptococcaceae, Lachnospiraceae	Firmicutes 56.643% ± 10.453%, Bacteroidetes 36.329% ± 11.369%, Verrucomicrobia 0.764% ± 0.579%, Proteobacteria 3.139% ± 1.969%, Fusobacteria 2.273% ± 2.270%, Actinobacteria 0.818% ± 0.307%, Firmicutes/Bacteroidetes ratio 15.568 ± 10.511. Acidaminococcaceae 9.886% ± 4.001%. Sutterellaceae, 0.429% ± 0.128%, Desulfovibrionaceae, 0.174% ± 0.116%. Enterococcaceae 0.001% ± 0.001%, Leuconostocaceae 0.000% ± 0.000%, Clostridiaceae 0.013% ± 0.007%, Peptostreptococcaceae 0.056% ± 0.024% Lachnospiraceae 9.798% ± 2.935%	Firmicutes 72.796% ± 4.936%, Bacteroidetes 20.446% ± 4.866%, Verrucomicrobia 1.805% ± 1.713%, Proteobacteria 0.507% ± 0.133%, Fusobacteria 0.364% ± 0.184%, Actinobacteria 4.074% ± 1.471%, Firmicutes/Bacteroidetes ratio 43.781 ± 33.594. Acidaminococcaceae 0.387% ± 0.211%. Sutterellaceae, 0.040% ± 0.015%, Desulfovibrionaceae, 0.052% ± 0.035%. Enterococcaceae 0.054% ± 0.026%, Leuconostocaceae, 0.093% ± 0.060%, Clostridiaceae 2.134% ± 1.311%, Peptostreptococcaceae 7.782% ± 5.631%, Lachnospiraceae 34.397% ± 4.899%,	Acidaminococcaceae *p* = 0.037, Sutterellaceae *p* = 0.007, Desulfovibrionaceae *p* = 0.020, Leuconostocaceae *p* = 0.004, Clostridiaceae *p* = 0.003, Peptostreptococcaceae *p* = 0.020, Lachnospiraceae *p* = 0.009
Liu et al. [64]		Flow cytometry-based IgA1 bacterial cell sorting, 16S rRNA gene sequencing (IgA-SEQ)	44 IBS-D, 32 healthy control	IBS-D	IgA+ bacteria	IgA+ cells s in the terminal ileum 28.97 6 ± 14.20 cells/hpf. Percentage of fecal IgA+ bacteria 53.44 6 ± 19.35. High relative abundance IgA+ bacteria: *Escherichia–Shigella, Romboutsia, Streptococcus*, *Tyzzerella_4*, *Klebsiella, Intestinibacter, Granulicatella*, and *Haemophilus* in IgA+ bacteria compared with those in IgA− bacteria. IgA+ *Escherichia–Shigella* 6.346% ± 6 12.01%,	IgA+ cells s in the terminal ileum 20.93 6 ± 10.27 cells/hpf, percentage of fecal IgA + bacteria 31.09% ± 6 17.31%. IgA+ *Escherichia–Shigella* 0.4814 ± 6 0.6319%	IgA+ cells s in the terminal ileum *p* = 0.0641, BAFF-R expression *p* = 0.0003, TACI *p* = 0.0769. Percentage of fecal IgA+ bacteria *p* = 0.0024, IgA+ *Escherichia–Shigella p* = 0.003199
Chen et al. [2]		Illumina high-throughput sequencing	20 IBS-D, 20 healthy volunteer	IBS-D	Community abundance: Lachnospiraceae UCG-004, Prevotella, Megamonas, Fusobacterium, Parabacteroides, Flavonifractor, Phascolarctobacterium	Community abundance: Lachnospiraceae UCG-004 20.96%, Prevotella 9 10.62%, Megamonas 12.74%. Fusobacterium, Parabacteroides, Flavonifractor, Phascolarctobacterium, unclassified Lachnospiraceae, and *Parasutterella* showed significantly different abundances between the stool specimens of the IBS group and normal group. Phascolarctobacterium and *Parasutterella* were positively related to the subcutaneous tissue inflammatory cells/epithelial cell number ratio	Community abundance: Lachnospiraceae UCG-004 12.97%, Prevotella 9 20.05%, Megamonas hardly found	mRNA levels of TLR-4 *p* = 0.013, TLR-5 *p* < 0.001. mRNA levels of IL-6*P* = 0.003, CXCL-11 *p* < 0.001, CXCR-3 *p* < 0.001. Upregulated expression of IL-6 (*p* = 0.002), CXCL 11 *p* < 0.001, CXCR 3 (*p* < 0.001). IL-10 (*p* = 0.012)
Shukla et al. [35]		Quantitative real-time polymerase chain reaction, immunohistochemistry	47 IBS (20 IBS-C, 20 IBS-D, 7 IBS-U), 25 controls	IBS-C, IBS-D, IBS-U	mRNA levels of TLR-4, TLR-5, IL-6, CXCL 11, CXCR-3-1	mRNA levels of TLR-4 median 0.24 (0.0–26.6) increased 4.2-folds, TLR-5 0.77 (0.0–6.5) increased 6.6 folds. mRNA levels of IL-6 0.90 (0.0–5.0), CXCL-11 2.80 (0.0–98.8), CXCR-3 1.80 (0.0–24.8). Upregulated expression of IL-6, CXCL 11, CXCR 3. IL-10 downregulated	mRNA levels of TLR-4 median 0.03 (0.0–13.1), TLR-5 0.03 (0.0–1.5). mRNA levels of IL-6 0.10 (0.0–2.8), CXCL-11 0.10 (0.0–15.4), CXCR-3 0.00 (0.0–25.2)	Copy number of *Lactobacillus* (*p* = 0.045) and *Bifidobacterium* (*p* = 0.011) showed correlation with IL-10 in IBS-C, while Gram-positive (*p* = 0.031) and Gram-negative bacteria (*p* = 0.010) showed correlation with CXCL-11 in IBS-D patients
Sun et al. [74]	Feces	16S rRNA sequencing	162 IBS-D, 66 healthy controls	IBS-D	Prevotellaceae, genus of Prevotella 9, Prevotella 2, Alloprevotella, *Escherichia–Shigella*, norank f, Lachnospiraceae, Ruminococcaceae UCG-013 in male groups and genus of Prevotella 2 in female groups	Significant increase in Prevotella 9 and *Escherichia–Shigella* in IBS-D (male). Bacteroides decreased	Significant decrease in Prevotella 9 and *Escherichia–Shigella* in controls (male)	Prevotella, *p* = 0.025 for IBS, *p* = 0.026 for control. Bacteroides, *p* = 0.079

The Unclassified genera *Eggerthella*, *Lactococcus, Turicibacter*, and *Fusobacterium* were more abundant, whereas *Coprococcus, Lachnobacterium, Faecalibacterium, Ruminococcus*, and *Akkermansia* were less abundant [23]. Additionally, the Firmicutes/Bacteroidetes (*F*/*B*) ratio was high when compared to healthy controls [23].

The bacteria associated with low-grade inflammation in the gut included increased *Actinomyces, Rothia, Gemella, Streptococcus, Enterococcus, Granulicatella, Fusobacterium, Escherichia/Shigella, Veillonella*, *Clostridium* cluster XI, and decreased *Anaerostipes, Fusicatenibacter, Odoribacter, Oscillibacter, Roseburia, and Faecalibacterium* [34].

Furthermore, another study determined significantly higher abundance of Acidaminococcaceae, Sutterellaceae, Desulfovibrionaceae, and substantially lower growth of Enterococcaceae, Leuconostocaceae, Clostridiaceae, Peptostreptococcaceae, and Lachnospiraceae in those with IBS than in control group participants [27].

Fungal phyla Zygomycota and four fungal markers, *Mycosphaerella, Aspergillus, Sporidiobolus,* and *Pandora,* were found in lower abundance in individuals with IBS compared to healthy controls [23]. Additionally,  bacterial/fungal interactions were altered, showing a reduction  in individuals with IBS compared to healthy controls [23].

**Fig. 9:**
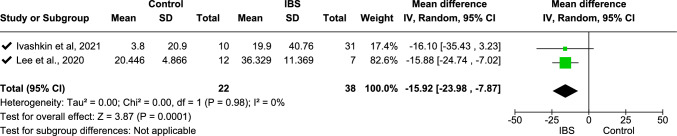
Meta-analysis on Bacteroidetes between people with IBS and healthy control participants

**Fig. 10:**
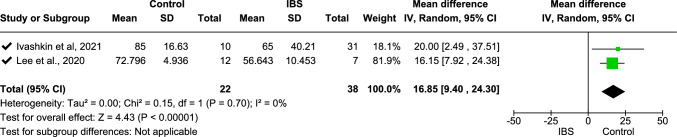
Meta-analysis on Firmicutes between individuals with IBS and healthy control participants

Meta-analysis was undertaken for the relative abundance of Bacteroidetes and Firmicutes, with data from two studies included for both [25, 27]. From the meta-analysis, Bacteroidetes were significantly more abundant [mean difference − 15.92 (− 23.98, − 7.87), *p* < 0.0001] in individuals with IBS. In contrast, Firmicutes were significantly more abundant in control group participants [mean difference 16.85 (9.40, 24.30), *p* < 0.00001]. Using mean difference with random effect model analysis, *I*^2^ = 0 with overall effect test *Z* = 3.87 (*p* = 0.0001) for Bacteroidetes (Fig. 9) and overall effect test *Z* = 4.43 (*p* < 0.00001) for Firmicutes (Fig. 10).

This forest plot shows the relative abundance of Bacteroidetes between people with IBS and healthy controls. Pooled standardized mean differences (SMDs) indicate a significantly higher relative abundance of Bacteroidetes in those with IBS (SMD − 15.92 [− 23.98, 7.87], *p* < 0.00001) with no heterogeneity (*I*^2^ = 0%).

This forest plot shows the relative abundance of Firmicutes between people with IBS and healthy controls. Pooled standardized mean differences (SMDs) indicate a significantly lower relative abundance of Firmicutes in those with IBS (SMD 16.85 [9.40, 24.30], *p* < 0.00001) with no heterogeneity (*I*^2^ = 0%).

### Linking Low-Grade Inflammation with Gut Microbial Changes

Three studies combined analysis of inflammatory markers and the gut microbiota [25, 35, 74]. Increased inflammatory markers were correlated with the changes in the gut bacteria (Table 6).

**Table 6:** Studies combining inflammatory markers and gut bacteria

Author, year	Inflammatory marker	Gut bacteria
Ivashkin et al., 2021 [25]	Increased TNFα, IL-2	Increased Bacteroidetes
Shukla et al., 2018 [35]	Increased TLR4, TLR5, IL-6, CXCL-11	Gram-positive and gram-negative bacteria (*p* = 0.010) showed a correlation with CXCL-11 in IBS-D patients
	Decreased IL-10	Copy number of *Lactobacillus* (*p* = 0.045) and *Bifidobacterium* (*p* = 0.011) showed correlation with IL-10 in IBS-C
Sun et al., 2021 [74]	Increased IL-12	Increased Prevotella 9 and *Escherichia–Shigella* in IBS-D (male), decreased Bacteroides

### Quality Assessment

**Fig. 11:**
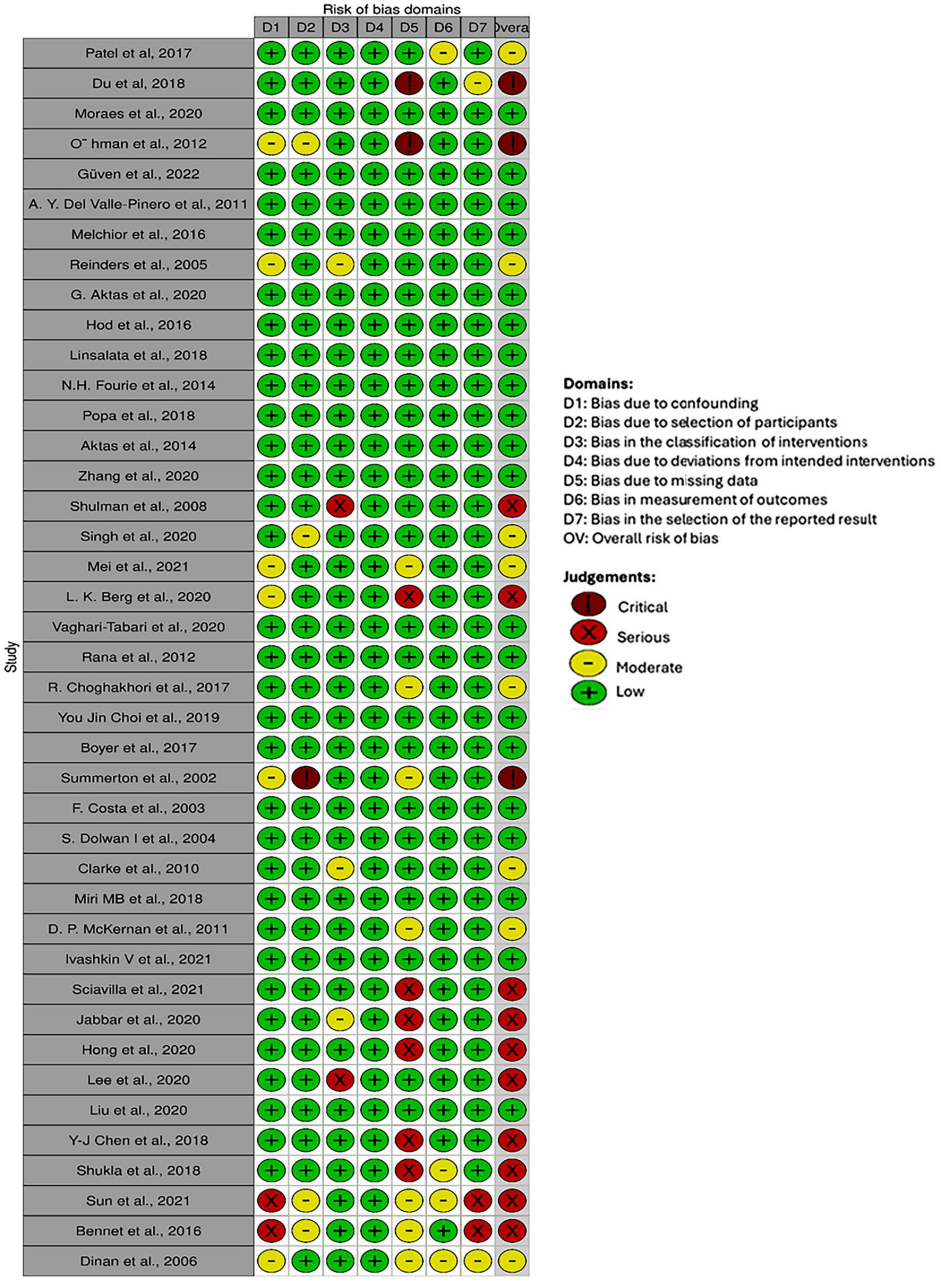
Risk of bias assessment by individual authors of the included studies for each domain

For the qualitative assessment, the ROBINS-I (Risk of Bias in Non-randomized Studies: of Interventions) method by Cochrane was followed and visualized with the ‘robvis’ tool. Of the 41 studies, 20 were low risk, 8 were moderate risk, 10 were serious risk, and 3 were critical risk of bias (Fig. 11). Two of the critical risk studies lacked complete data [21, 32] and one lacked detailed information about the participants [18] (Fig. 11). Although there was a lack of complete data in one study, there were data on inflammatory markers that met the inclusion criteria and were included in the meta-analysis; hence, the study was not excluded [32].

**Fig. 12:**
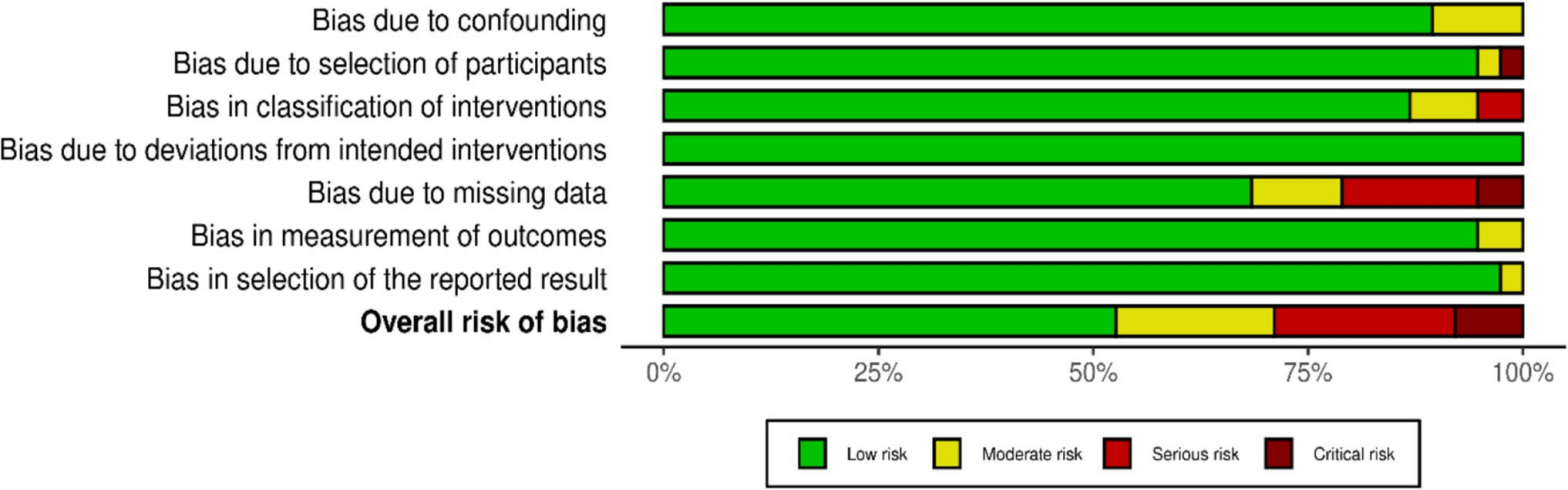
Collective risk of bias assessment for all the included studies for the systematic review

Among the ten serious risk studies, nine studies lacked proper data and reporting[2, 14, 26, 35, 74, 75] (none of which were included in the meta-analysis), while two studies showed bias in the classification of interventions [27, 36]. Among eight moderate-risk studies, two studies showed bias in the measurement of outcomes [11, 71], three studies showed bias due to missing data [30, 64, 71] (not included in the meta-analysis), two studies showed bias in the classification of interventions [19, 60], and one study showed bias in the selection of the participants [37]. Overall, most of the studies had a low risk of bias, with some notable risk of bias in missing data and the selection of the classification of interventions (Fig. 12).

## Discussion

This systematic review, based on 41 research articles, presents evidence of increased levels of various inflammatory markers and altered gut microbiota in individuals with IBS. The included studies were conducted in multiple countries and investigated a range of direct and indirect inflammatory biomarkers that were significantly distinguishable between those with IBS and control participants without any inflammation. However, there were inconsistencies between studies that reported findings for the same cytokine. For example, IL-10 was reportedly reduced in IBS compared to healthy individuals in four studies [11, 14, 16, 33], whereas levels were reportedly increased in one study [32]. One explanation is that different cohorts were investigated, with some studies dominated by participants displaying a T_H_ type 1 phenotype (associated with decreased IL-10), and the conflicting study dominated by participants displaying a T_H_ type 2 phenotype (associated with increased IL-10) [24]. There is evidence that IBS may be associated with a particular T_H_ type phenotype, shown by a positive correlation of IL-1β, TNF-α, and IL-6, and a negative correlation of IL-10 and IL-12 with IBS symptoms [24]. Furthermore, alleles associated with excessive production of IL-6, IL-2, and TNF-α and decreased production of IL-10 are more frequently found in IBS [11] The rate of IL-10 polymorphisms is higher in people with IBS than in healthy controls [77].

There is also evidence of alterations in non-cytokine inflammatory markers in IBS. TLRs, which can stimulate pro-inflammatory cytokine production [32, 65], were reportedly higher in IBS (in terms of both gene expression and plasma concentrations/protein) [28, 35], although this did not correlate with IBS symptoms. However, TLR expression was correlated with less severe psychological symptoms among people with IBS, offering immunological parameters for symptom generation in a subgroup of IBS [32].

In all studies, fecal calprotectin was found to be considerably higher in people with IBS than in control participants, suggesting it may be useful in the diagnosis of IBS [62]. However, fecal calprotectin is routinely used to diagnose inflammatory bowel disease (IBD) and can distinguish between IBS and IBD with high diagnostic accuracy [78]. In most cases, faecal calprotectin levels are usually similar or slightly higher in IBS than in controls. While diagnosing IBS, different studies used the detection of fecal calprotectin [79]. One study investigated 93 adults with IBS and delineated a subgroup of 34 with higher fecal calprotectin [73]. The only difference between the two groups was age: those with high calprotectin tended to be older than those with normal calprotectin. The study did not find any correlation between the elevated calprotectin and other pathophysiological conditions (confirmed by fructose and glucose breath test, rectal hypersensitivity, and histological colonic inflammation) [73]. Although fecal calprotectin levels are not consistently elevated in IBS patients, the test remains a valuable tool for diagnosing IBD and differentiating it from IBS.

Another potential diagnostic marker for IBS is RDW, which was significantly higher in the setting of IBS when compared with controls (*p* < 0.001) [60]. MPV may also be a useful marker in making a diagnosis of IBS [60, 69]. As one study suggested, both RDW and MPV are potential diagnostic biomarkers [60]; another study aimed to use these biomarkers to distinguish between IBS, IBD, and healthy controls [69]. These two studies showed different outcomes for MPV. One study detected a slight MPV increase, and the other showed a slight decrease in MPV. Both studies suggested the need for further investigation with a larger cohort to confirm the validity of the results.

Beyond blood-based biomarkers, local immune responses in the gut also play a crucial role in IBS pathology. For instance, mast cells can induce low-grade inflammation by producing pro-inflammatory cytokines upon degranulation [13, 37]. In two studies, among the people with IBS, mast cells were shown to have greater density close to enteric nerves, with a relationship to abdominal pain severity (*r* = 0.75, *p* = 0.001 and *r* = 0.70, *p* = 0.003) [13, 37]. Mast cells secrete mediators that alter the enteric nerve and smooth muscle functionality, which may contribute to IBS symptoms (abdominal pain, constipation, and diarrhea) [43]. Mast cells can be activated in a variety of ways, including food antigen, allergen, commensal bacteria and their by-products, toxins, and psychological distress [80].

In a small number of studies, eosinophils were shown to be increased in the setting of IBS, with a correlation between the increase in mast cell activation and eosinophil density [15, 67, 81].

Similarly, hsa-miR-150 and hsa-miR-342-3p were measured by one study [63]. Elevated levels of hsa-miR-150 are related to pain severity in IBD, and hsa-miR-342-3p is linked with smooth muscle function, pain signaling, and colonic motility [63] and, thus, may be associated with IBS symptoms.

Additionally, NLR, MLR, SII, PLR, macrophages, neutrophils, T-lymphocytes, MCP-1, MIP-1α, MIP-1β, RANTES, CD3+  T cells, and IELs were increased in the setting of IBS compared to control [7, 12, 14, 67]. The higher numbers of CD3+  T cells and IELs play a crucial role in immunopathology [67]. Moreover, one study showed a significant increase in MDA (*p* < 0.05) and a significant decrease in TAC (*p* < 0.05) [16], but these results failed to show any correlation with disease symptoms or quality of life [16]. Cortisol is a hormone produced from the hypothalamic–pituitary–adrenal (HPA) axis that indicates stress [25]. Changes in the stimulation of cortisol are responsible for immune function dysregulation, resulting in the production of inflammatory cytokines [65, 82]. Serum levels of cortisol were found to be increased in people with IBS, and this cortisol was also correlated with increased IL-6 [65, 68]. This correlation highlights the mechanism by which stress triggers the hormonal changes by producing more cortisol, which can trigger inflammatory responses in IBS. It is possible for targeted therapies like managing stress (therapies like cognitive–behavioral therapy, CBT) and low-grade inflammation (anti-inflammatory medicines) to control the symptoms of IBS [83, 84].

AA, PGE_2_, and LTB4 were reported to be increased in IBS in one study [19]. However, levels of these biomarkers were not correlated with the severity of symptoms. PGE_2_ can elevate the inflammatory cytokine IL-6, though this becomes insignificant as it is immediately changed into a more stable PGH_2_ form [19, 85]. LTB4 can promote functional and morphological changes in mast cells that have been observed in IBS and might be related to the inflammatory degranulation of mast cells, indirectly contributing to the development of symptoms [13].

Furthermore, NO is higher in IBS compared to healthy controls, with increased NO detected for all functional low-grade gastrointestinal inflammation [61]. This suggests that there is an inflammatory response in IBS [86]. Moreover, one study showed a significant increase in growth factors bFGF, PDGFBB, and GM-CSF [14]. Generally, the role of growth factors is to maintain cellular integrity and impart an anti-inflammatory effect, which is observed in mucosal healing in IBD [87]. The increase in these growth factors may indicate the presence of mucosal damage in IBS.

Another inflammatory marker found to be significantly elevated in IBS is PRDX1. PRDX1 is a nonspecific inflammatory marker that was first found to be higher in studies of people with ulcerative colitis, UC [88]. PRDX1 is co-activated with TLR2 and TLR4 and shows increased expression in association with symptom severity in IBS-D [70, 89]. PRDX1 correlates with IBS symptoms and, therefore, may have utility as a diagnostic marker. Additionally, CCL-16 gene expression promotes the production of inflammatory biomarkers, and this was confirmed by a study reporting 7.46-fold higher production in IBS compared to healthy controls [3]. Furthermore, CCL-16 was overexpressed 130-fold in the IBS-C subtype when compared with healthy controls and IBS-D in the same study [3, 90]. However, another study showed a decrease in CCL-16 protein between IBS and the control group, with a significantly higher CCL-16 in people with IBS-D when compared with the IBS-M subtype [91]. An increase in the expression of the CCL-16 gene in IBS was detected in a previous study [3]. While most current studies indicate elevated CCL-16 levels in individuals with IBS, the findings are not consistently statistically significant [3, 91, 92]. One study reported a significant elevation (*p* = 0.004) [91], whereas others found no significant difference compared to healthy controls [3, 93]. These discrepancies may stem from methodological differences, including variations in how CCL-16 was measured (gene expression vs. protein level), differences in study populations (e.g., subtype distribution vs. no subtyping), or small sample sizes. Despite the inconsistent findings, the pattern of altered CCL-16 expression in different IBS subtypes is promising and warrants further investigation.

The IBS gut bacterial composition was also found to be altered in IBS, with a significant decrease in *Firmicutes, Actinobacteria*, and *Fusobacteria* and a significant increase in *Proteobacteria* and *Bacteroidetes* [25, 30]. However, several studies showed opposite results for *Firmicutes* and *Bacteroides* [48, 94, 95]. Variations observed between studies conducted in China and the United States suggest that regional and dietary factors may contribute to the differing results [30]. The lower levels/amounts/abundance of *Firmicutes* (short-chain fatty acid producers) in IBS interrupts the production of SCFAs, weakening the epithelial barrier [25]. This leads to the pathophysiology of IBS (for example, altered gut permeability, immune activation, and symptom aggravation) [25].

A higher abundance of *Bacteroidetes* can degrade mucosal protein and damage cellular integrity [25, 96]. Increased *Proteobacteria* can initiate low-grade inflammation in the gut that can contribute to IBS symptoms [30]. Furthermore, *Enterobacteriaceae, Streptococcus, Fusobacteria, Gemella*, and *Rothia*, reported to be associated with low-grade gut inflammation, were found to be more abundant in the setting of IBS than in healthy controls [34]. Meanwhile, reportedly beneficial bacteria (Roseburia and Faecalibacterium) were found to have lower abundance in IBS [34]. Furthermore, increased IgA+-coated bacteria *Escherichia–Shigella, Granulicatella, and Haemophilus* were found in IBS-D compared to healthy controls by the activation of IgA class switching [64]. This could modulate local low-grade inflammation, steering the clinical manifestation of IBS-D [64].

*Parasutterella* has also been associated with low-grade intestinal inflammation, similar to *Escherichia–Shigella*, which suggests a potential role in promoting inflammation within the gut microbiota [2]. A positive relationship between the amount of *Parasutterella* bacteria and the ratio of inflammatory cells to epithelial cells in subcutaneous tissue has been observed, suggesting that *Parasutterella* could be associated with subclinical intestinal inflammation in people with IBS [2].

Moreover, a reduction in *Lactobacillus* and *Bifidobacterium* has been associated with low-grade inflammation in IBS [35]. *Lactobacillus* has been shown to stimulate mucin expression, which may help enhance epithelial tight junctions [97]. The potential for *Lactobacillus* as a probiotic therapy in IBS is promising, as it could have anti-inflammatory and immunomodulatory effects [97]. Both *Lactobacillus* and *Bifidobacterium* produce short-chain fatty acids (SCFAs), which help protect the intestinal lining and reduce the ability of pathogenic bacteria to adhere [98–100]. A decrease in these bacteria may allow pathogenic bacteria to adhere more readily and produce excessive gas through carbohydrate fermentation, potentially contributing to gastrointestinal symptoms such as discomfort and bloating, which are commonly associated with IBS [98–100].

In addition to inflammation and dysbiosis, key physiological mechanisms such as increased intestinal permeability, impaired epithelial barrier function, and reduced SCFA production have emerged as important mediators in IBS. SCFAs like butyrate play a critical role in maintaining mucosal integrity by enhancing epithelial tight junctions and suppressing inflammation [101, 102]. A deficiency in SCFA-producing bacteria, such as *Lactobacillus* and *Bifidobacterium*, may compromise this barrier, increase intestinal permeability, and facilitate the translocation of microbial products, like LPS, across the epithelium [101–103]. Despite their central role, these factors were not consistently assessed across the included studies, highlighting an important area for future research.

Furthermore, during this systematic review, *Brachyspira* was found in 40% of [26] cases of IBS-D in a study, suggesting that its potential role in the pathogenesis may be relevant for a subset of IBS patients. The *Brachyspira* genus includes pathogenic bacteria that are associated with intestinal spirochetosis, a condition characterized by symptoms like diarrhea and abdominal pain [26]. During spirochetosis, *Brachyspira* penetrates the mucus layer and colonizes the colonocyte apical membrane. Total and activated mast cells were significantly more abundant in patients with IBS with *Brachyspira* but not in patients with IBS without *Brachyspira* [26]. This indicates that *Brachyspira* may be associated with low-grade inflammation and symptom severity in IBS-D.

Gut microbes are closely associated with the immune system and emotional regulation via the gut–brain axis, BGA [104]. In normal circumstances, innate immunity helps maintain microbial homeostasis. However, dysbiosis, which can be triggered by factors such as infection, dietary changes, or stress, may disrupt this balance and has been associated with low-grade inflammation through the activation of TLR-mediated cytokine release (e.g., IL-6, IL-10) [25, 35, 98]. Alterations in the microbial community may also lead to changes in the production of microbial metabolites, weakening of the gut barrier, and allowing the translocation of bacterial products like LPS. This can potentially activate macrophages and contribute to low-grade inflammation [105–107].

Furthermore, the bidirectional gut–brain axis (BGA) connects the microbiome to neural, endocrine, and immune responses, potentially influencing gut motility, pain, and mood [76, 108]. Certain bacteria have been shown to affect neurotransmitter levels—*Bifidobacterium dentium*, for instance, produces GABA, and its presence may be linked to improvements in depressive symptoms in IBS [108, 109]. Associations have also been observed between Acidaminococcaceae and anxiety, and Peptostreptococcaceae with changes in brain structure [27, 105], although the role of Sutterellaceae in these processes remains unclear [27].

**Fig. 13:**
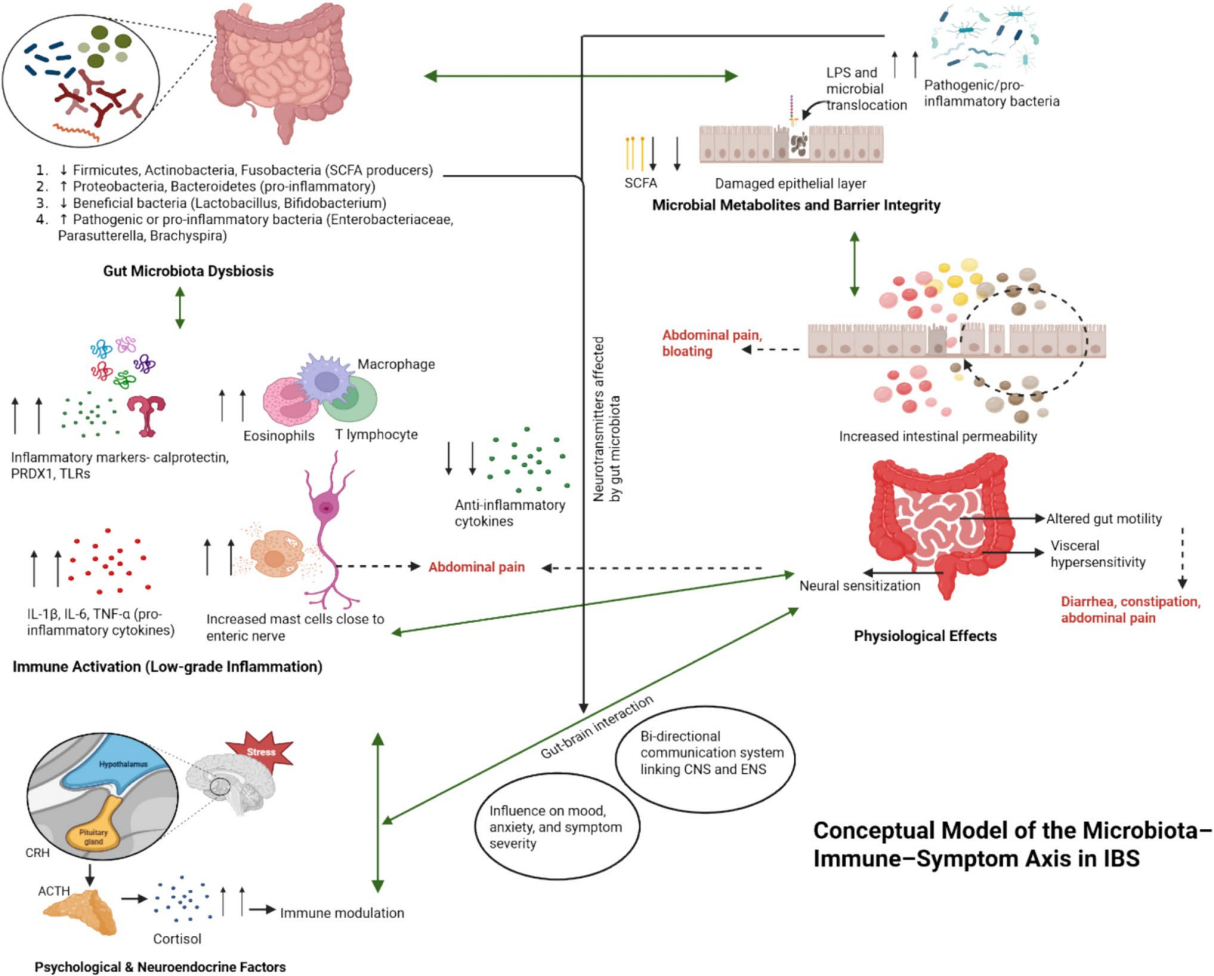
Conceptual model of the hypothesized microbiota–immune–symptom axis in irritable bowel syndrome (IBS), developed from studies included in this systematic review. **↑↑ increase, ↓↓ decrease, *CRH* corticotropin-releasing hormone, *ACTH* adrenocorticotropic hormone

To consolidate the complex interplay between microbial shifts, immune activation, and symptom manifestation in IBS, a schematic model (Fig. 13) was developed based on the studies included in this review. The model summarizes interconnected pathways identified across the included studies. Gut microbiota dysbiosis—characterized by decreased SCFA-producing bacteria (e.g., *Firmicutes*, *Lactobacillus*, *Bifidobacterium*) and increased pro-inflammatory taxa (e.g., *Proteobacteria*, *Enterobacteriaceae*)—can compromise epithelial barrier integrity, leading to increased intestinal permeability and translocation of microbial products such as lipopolysaccharide (LPS). This stimulates immune responses via Toll-like receptors (TLRs), mast cells, and cytokine production (e.g., IL-6, IL-1β, TNF-α), contributing to low-grade inflammation. Inflammatory signals interact with the enteric nervous system, influencing visceral sensitivity, motility, and pain perception. Psychological stress further modulates these pathways via the hypothalamic–pituitary–adrenal (HPA) axis, increasing cortisol levels that may exacerbate immune dysregulation. These overlapping mechanisms may form a bidirectional loop contributing to the onset or persistence of IBS symptoms.

The limitation of this systematic review is that most of the studies did not recruit age and gender-matched participants (IBS and control). Also, there were significant differences in the number of participants from each group. While recruiting healthy participants for the control group, some studies considered dietary elements [2, 15, 23, 28, 34] and psychiatric conditions [68], while others did not. Variation in such conditions of recruited participants leaves scope for better statistically significant results with broader inclusion of the studies in the meta-analysis.

Another important limitation lies in the methodological heterogeneity across the included studies. Most of the studies focused only on a single subtype, while the rest of the studies did not distinguish between different IBS subtypes, making comparisons and generalizations more difficult. Furthermore, microbiota analysis methods varied significantly—from 16S rRNA sequencing to metagenomic approaches—potentially affecting consistency in microbial identification and interpretation. Psychological comorbidities such as anxiety, depression, and stress were assessed in some studies but not in others. These differences across study design and participant characterization contribute to heterogeneity in the findings and may have influenced the overall conclusions of this systematic review.

## Conclusion

This review provides evidence from various studies suggesting that microbial dysbiosis may contribute to changes in intestinal cellular integrity, symptom severity, immune activation, and low-grade gut inflammation in the context of IBS. Additionally, there is evidence indicating a positive association between altered gut microbiota and low-grade inflammation with anxiety and depression, which are commonly observed in IBS. Reduced levels of beneficial bacteria in IBS may create an environment conducive to gut microbial dysbiosis, potentially disrupting the normal physiological functions of the gut. The increased production of inflammatory cytokines, associated with changes in gut bacterial composition, also appears to correlate with IBS symptoms. However, many studies focusing on microbial changes and inflammatory markers in IBS emphasize the need for further research with larger populations to confirm the role of low-grade inflammation and microbial shifts. While additional studies are necessary, the factors contributing to IBS are complex and interrelated. Identifying and characterizing these factors in individuals with IBS could be essential for developing more effective treatment strategies.

## Supplementary Information

Below is the link to the electronic supplementary material.

Supplementary file1 (XLSX 33 KB)

Supplementary file2 (XLSX 12 KB)

## Data Availability

Raw data extraction files have been provided as supplementary materials. No additional data were generated during the study. **Consent for Publication** Consent from participants for publication is not required, as no individual participant information was used in the systematic review. However, all authors of this article have read and approved the final manuscript and consent to its submission for publication.
